# A multicenter prospective validation cohort does not support the use of kidney/psoas [^18^F]FDG uptake in the diagnosis of kidney allograft subclinical rejection

**DOI:** 10.1371/journal.pone.0345426

**Published:** 2026-09-02

**Authors:** Pierre Lovinfosse, Antoine Bouquegneau, Annick Massart, Lissa Pipeleers, Catherine Bonvoisin, Laurens Carp, Hendrik Everaert, Alexandre Jadoul, Amélie Dendooven, Caroline Geers, Stéphanie Grosch, Pauline Erpicum, Rachel Hellemans, Laurence Seidel, Laurent Weekers, Roland Hustinx, François Jouret

**Affiliations:** 1 Division of Nuclear Medicine, Department of Medical Physics, University of Liège Hospital, Liège, Belgium; 2 GIGA Institute, in vivo Imaging, Liège, Belgium; 3 Division of Nephrology, Department of Internal Medicine, University of Liège Hospital, Liège, Belgium; 4 GIGA Institute, Metabolism & Cardiovascular Biology, University of Liège, Liège, Belgium; 5 Division of Nephrology, Department of Internal Medicine, Universitair Ziekenhuis Antwerpen, Antwerp, Belgium; 6 Department of Nephrology and Arterial Hypertension, Universitair Ziekenhuis Brussel, Vrije Universiteit, Brussels, Belgium; 7 Kidney Diseases, Dialysis & Transplantation Research Unit (NIER), Vitality Research Group, Vrije Universiteit, Brussels, Belgium; 8 Division of Nuclear Medicine, Universitair Ziekenhuis Antwerpen, Antwerp, Belgium; 9 Division of Nuclear Medicine, Department of Medical Physics, Universitair Ziekenhuis Brussel, Brussels, Belgium; 10 Faculty of Health Sciences, University of Antwerp, Antwerp, Belgium; 11 Department of Pathology, university Hospital of Ghent, Ghent, Belgium; 12 Division of Renal Pathology, Universitair Ziekenhuis Brussel, Brussels, Belgium; 13 Division of Renal Pathology, Unilab, University of Liège Hospital, Liège, Belgium; 14 Faculty of Medicine and Health Sciences, Laboratory of Experimental Medicine and Paediatrics, Wilrijk, Belgium; 15 B-STAT, University of Liège Hospital, Liège, Belgium; University Putra Malaysia, MALAYSIA

## Abstract

**Background:**

Subclinical kidney allograft acute rejection (SCR) corresponds to “the unexpected histological evidence of acute rejection in a stable patient”. The diagnosis of SCR relies on surveillance biopsy. Positron emission tomography (PET/CT) after injection of F^18^-fluorodeoxyglucose ([^18^F]FDG) has been proposed as a non-invasive screening approach. In the present multicenter prospective study, we assess the diagnostic yield [^18^F]FDGPET/CT to rule out SCR in stable KTR at 3 months *post* KTx.

**Methods:**

From 01/2021–03/2025, we prospectively combined surveillance biopsy and [^18^F]FDGPET/CT at ~3 months *post* transplantation in adult kidney transplant recipients from 4 independent imaging centers. The mean standardized uptake value (mSUV) was measured in kidney cortex and referenced as a ratio to psoas muscle mSUV (mSUVR).

**Results:**

Our multicenter cohort of 185 patients was categorized according to the Banff-2022 classification as: normal (n = 158); borderline (n = 18); SCR (n = 9, including 6 T-cell-mediated rejection and 3 microvascular inflammation). No significant correlation was observed between the mSUVR and ti score (R = 0.032, p-value = 0.67). The mSUVR reached 2.33 [1.97–2.93], 2.71 [2.50–3.33] and 2.42 [2.27–3.14] in normal, borderline and SCR groups, respectively. In multivariate models stratified by center, the risk of non-normal histology (n = 27, including borderline and SCR) increased with donor age (OR=1.05 [1.01–1.1], p = 0.02) but not with the mSUVR (OR=4.11 [0.91–18.48], p = 0.07). The Z-score of mSUVR was significantly associated with the risk of non-normal histology (OR=1.542 [1.02–2.33, p = 0.04). The risk of biopsy-proven SCR (n = 9) was not significantly associated with mSUVR.

**Conclusions:**

The mSUVR of [^18^F]FDG PET/CT does not reliably rule out SCR on surveillance biopsy.

## Introduction

The systematic follow-up of kidney transplant recipients (KTRs) is essential to early detect transplant rejection and appropriately personalize the immunosuppressive regimen [1]. The diagnostic gold-standard for allograft rejection relies on the Banff classification, which provides standardized histopathological criteria for the grading of allograft rejection, including the assessment of microvascular inflammation (MVI) [2]. Subclinical rejection (SCR) has been defined as ‘the documentation by light histology of unexpected evidence of allograft rejection in a stable patient’ [3]. Surveillance transplant biopsies are, by definition, required for the diagnosis of SCR [4]. Although an ultrasound-guided core needle biopsy of kidney allograft is regarded as relatively safe, it remains an invasive procedure with a ~ 7% rate of complications [5]. Furthermore, inter-observer variability and sampling errors limit its benefits. In order to optimize the currently indiscriminate use of allograft biopsies in stable KTRs, non-invasive approaches could be developed as “rule out” tests, with the highest negative predictive value [6–8]. Various non-invasive modalities for the diagnosis of SCR are currently under investigation, including imaging, gene expression profiling and omics analyses of blood and urine samples [9–11]. We and others have suggested that ^18^F-Fluorodeoxyglucose Positron Emission Tomography coupled with Computed Tomography ([^18^F]FDGPET/CT) may help to non-invasively distinguish the absence of biopsy-proven acute rejection [12–15]. Hypothetically, the immunological reaction against donor antigens induces the recruitment of mononuclear leukocytes into the renal transplant, which basically corresponds to the core of the Banff classification. The boosted metabolism of these inflammatory cells could be assessed by PET quantification of the renal uptake of [^18^F]FDG. A renal mean standardized uptake value (mSUV) below a screening threshold would therefore suggest the absence of active inflammation in the allograft, thereby precluding the surveillance biopsy. In order to mitigate the impact of sensitivity disparities of various [^18^F]FDGPET/CT devices, “target-to-background” ratios can be used. On the basis of a pilot prospective monocentric 92-KTR cohort, we postulated that a ratio of kidney/psoas mSUVR lower than 2.4 would non-invasively exclude SCR, with a negative predictive value of 98% [16]. In the present multicenter validation study, we prospectively assess the diagnostic yield of this ≤2.4 mSUVR threshold to rule out SCR in stable KTR at 3 months *post* KTx.

## Patients and methods

### Patients

From January, 1 2021 to March, 31 2025, we prospectively performed [^18^F]FDGPET/CTimaging in stable adult KTR who underwent surveillance transplant biopsy at ~3 months *post* KTx in 3 independent transplant centers: Universitair Ziekenhuis Antwerpen (UZA), Universitair Ziekenhuis Brussels (UZB), and Centre Hospitalier Universitaire de Liège (CHU Liège). At CHU Liège, [18F]FDGPET/CT images were acquired on 2 different machines (*see infra*). This study was systematically proposed to all stable adult KTR who underwent surveillance transplant biopsy at ~3 months *post* KTx. Patients with a probable polyomavirus nephropathy (BK polyomavirus viremia > 3 log_10_ copies) and patients who underwent an ABO incompatible KTx were excluded. Written informed consent was systematically obtained. This study has been approved by the Institutional Review Board (IRB) of CHU Liège under the number B707202042977, and endorsed by the IRB of UZA and UZB. Kidney transplantations were performed in accordance with the Declaration of Istanbul. Our database has been registered as NCT03764124. Clinical, biological, histological and imaging parameters were systematically collected (Table 1).

**Table 1:** Characteristics of the cohort. Data are expressed as mean ± standard deviation; median [interquartile range]. AZA: Azathioprin; BMI, body mass index; DBD, donor after brain death; DCD, donor after circulatory death; DSA, donor-specific antibodies; eGFR, estimated glomerular filtration rate; FDG, fluorodeoxyglucose; KTx, kidney transplantation; LD, living donor; MMF, mycophenolate mofetil; MPA, mycophenolic acid; mTOR, mammalian target of rapamycin; PET/CT, positron-emission tomography/ computed tomography; PRA, panel reactive antigens.

	Global Cohort N = 185	Normal N = 158	Borderline N = 18	Rejection N = 9
**RECIPIENT AT BIOPSY**				
Age (years), mean ± SD	55.0 ± 12.8	55.0 ± 12.9	55.4 ± 9.31	53.7 ± 17.9
BMI (kg/m²), mean ± SD	25.7 ± 4.35	25.5 ± 4.46	27.2 ± 3.54	26.5 ± 3.17
Female sex, n (%)	67 (36.2)	60 (38.0)	5 (27.8)	2 (22.2)
PRA max (n (%))				
< 5%	140 (76.9)	120 (76.9)	14 (82.4)	6 (66.7)
5-85%	30 (16.5)	25 (16.0)	2 (11.8)	3 (33.3)
> 85%	12 (6.6)	11 (7.1)	1 (5.9)	0 (0.0)
**DONOR**				
Age (years), mean ± SD	48.4 ± 12.3	47.2 ± 12.5	55.9 ± 6.41	52.8 ± 12.4
BMI (kg/m²), mean ± SD	25.8 ± 4.62	25.8 ± 4.51	26.3 ± 5.55	25.1 ± 5.13
Female sex, n (%)	73 (39.7)	63 (40.1)	7 (38.9)	3 (33.3)
Type				
DBD	95 (51.4)	84 (53.2)	5 (27.8)	6 (66.7)
DCD	62 (33.5)	50 (31.6)	10 (55.6)	2 (22.2)
LD	28 (15.1)	24 (15.2)	3 (16.7)	1 (11.1)
**TRANSPLANTATION**				
Cold ischemia time (min), median [Q1-Q3]	421 [258 - 747]	421 [258 - 749]	388 [212 - 589]	480 [281 - 602]
Delayed graft function (n (%))	25 (13.5)	20 (12.7)	3 (16.7)	2 (22.2)
**STATUS AT TIME OF BIOPSY**				
Delay between KTx and biopsy (days), median [Q1-Q3]	91.0 [87.0 - 98.0]	91.0 [88.0 - 97.0]	88.5 [85.0 - 98.0]	94.0 [90.0 - 106]
Calcineurin Inhibitors (n(%))				
Tacrolimus	174 (94.1)	149 (94.3)	17 (94.4)	8 (88.9)
CsA	11 (5.9)	9 (5.7)	1 (5.6)	1 (11.1)
Anti-proliferative agents, n(%)				
None	7 (3.8)	5 (3.2)	2 (11.1)	0 (0.0)
MMF	174 (94.1)	149 (94.3)	16 (88.9)	9 (100.0)
AZA	4 (2.2)	4 (2.5)	0 (0.0)	0 (0.0)
mTOR Inhibitors (Yes (%))	6 (3.2)	5 (3.2)	1 (5.6)	0 (0.0)
Corticosteroids (Yes (%))	181 (97.8)	156 (98.7)	17 (94.4)	8 (88.9)
Creatinine (mg/dL), median [Q1-Q3]	1.31 [1.07 - 1.54]	1.28 [1.05 - 1.52]	1.44 [1.21 - 1.65]	1.46 [1.35 - 1.88]
Proteinuria (mg/g creat), median [Q1-Q3]	119 [81.5 - 190]	120 [81.5 - 190]	107 [80.0 - 183]	100 [87.4 - 205]
DSA (n(%))				
None	177 (96.2)	153 (97.5)	15 (83.3)	9 (100.0
Class I	3 (1.6)	1 (0.6)	2 (11.1)	0 (0.0)
Class II	4 (2.2)	3 (1.9)	1 (5.6)	0 (0.0)
**PET-CT**				
Blood glucose (mg/dL), median [Q1-Q3]	104 [93.0 - 120]	104 [93.0 - 122]	101 [92.0 - 115]	104 [97.5 - 112]
FDG activity (mBq), median [Q1-Q3]	222 [193 - 257]	220 [188 - 256]	237 [214 - 269]	257 [224 - 293]
Delay injection-PET (min), median [Q1-Q3]	180 [180 - 190]	180 [180 - 190]	180 [180 - 190]	180 [180 - 185]
Delay between biopsy and PET-CT (days), median [Q1-Q3]	0.51 [0.00 - 1.00]	0.51 [0.50 - 1.00]	−0.50 [−1.00 - 0.52]	0.51 [−1.00 - 1.00]
Delay between KTx and PET-CT (days), median [Q1-Q3]	91.5 [87.0 - 98.5]	91.8 [87.0 - 98.0]	88.5 [84.0 - 98.5]	95.0 [89.0 - 107]
**SUV**				
mSUV, median [Q1-Q3]	1.86 [1.68 - 2.16]	1.83 [1.64 - 2.11]	2.10 [1.84 - 2.20]	1.94 [1.82 - 2.26]
mSUVR, median [Q1-Q3]	2.37 [2.01 - 2.95]	2.33 [1.97 - 2.93]	2.71 [2.50 - 3.33]	2.42 [2.27 - 3.14]
**Z-scores of SUV**				
Z-mSUV, mean ± SD	0.00 ± 0.99	−0.045 ± 1.01	0.33 ± 0.78	0.11 ± 0.92
Z-mSUVR, mean ± SD	−0.00 ± 0.99	−0.076 ± 0.94	0.55 ± 1.26	0.23 ± 1.05

### Histopathology

**Fig 1:**
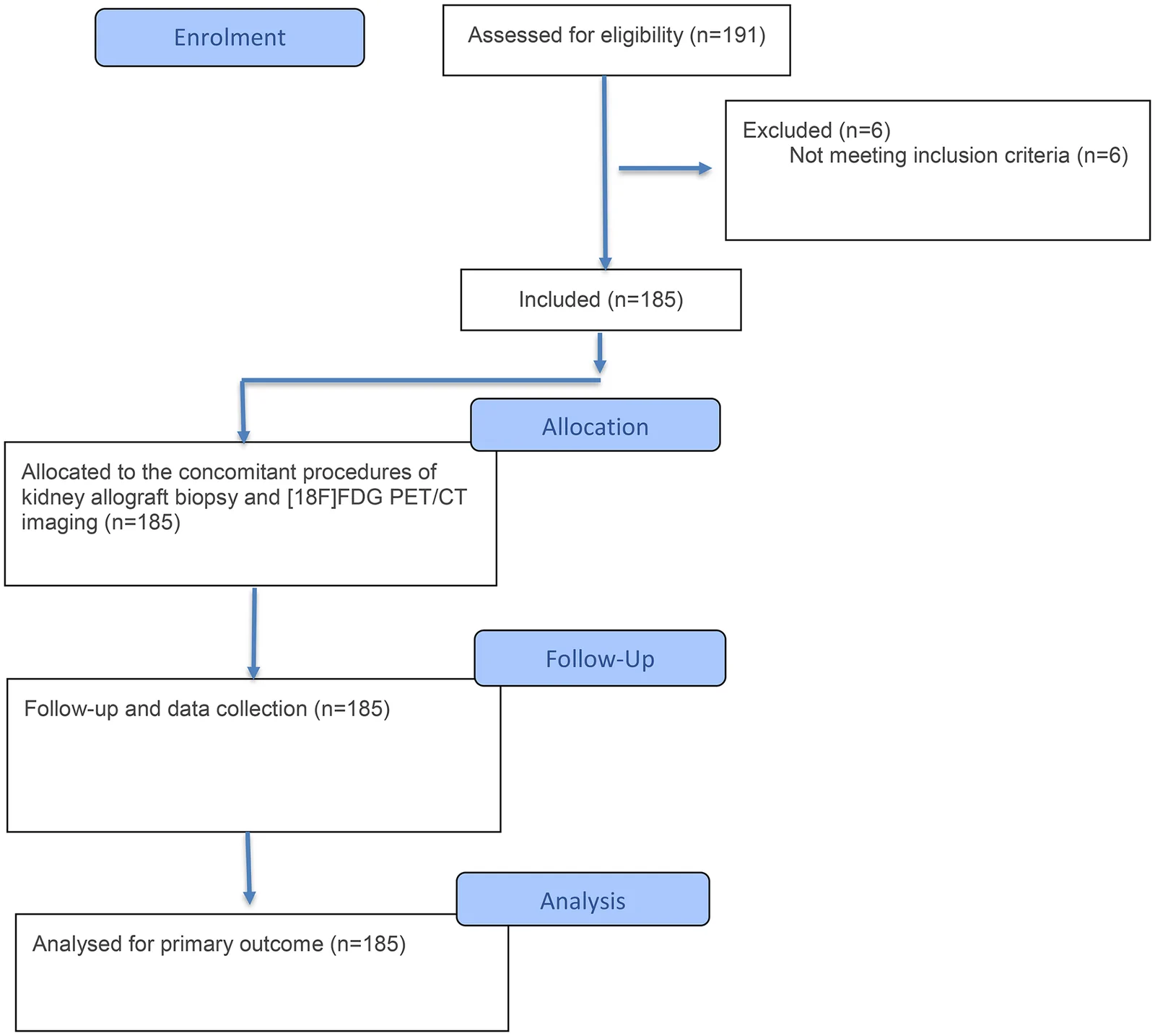
CONSORT 2025 Flow Diagram. Between January 2021 and March 2025, 191[^18^F]FDG PET/CT were prospectively performed at the time of surveillance transplant biopsy at ~3 months *post* transplantation. Six cases were excluded: 4 PCR-proven BK nephropathies and 2 uninterpretable histology.

Biopsies were assessed by pathologists blinded to the results of [18F]FDGPET/CT. Banff 2022 classification was applied. Acute and chronic histological lesions were scored from 0 to 3 on the basis of the severity of infiltration by mononuclear cells in each component (glomeruli (g), peritubular capillaries (ptc), arteries (v), tubules (t) and interstitium (i)). Total inflammation (ti) was scored considering interstitial inflammation in both non-sclerotic and sclerotic areas. C4d staining was performed in all cases. The Banff-based categorisation was performed using the raw results of the Banff 2022 classification, which were then integrated into the Banff Automation System for kidney allograft precision diagnostics [17]. This approach allowed us to obtain standardised results. Biopsies were classified into “normal”; “borderline” (corresponding to Banff category 3: suspicious for acute T-cell-mediated rejection (TCMR), defined by “i=1 and t≥1” or “i=2 and t=1-2”, or C4d staining without evidence of rejection) [2], or “acute rejection” (corresponding to Banff category 2: active antibody-mediated rejection (AMR), or Banff category 4: acute TCMR IA or higher). DSA-negative, C4d-negative MVI corresponded to “g+ptc ≥ 2; C4d (-); DSA (-)” [18]. The SCR group was defined as subclinical inflammation including biopsy-confirmed acute rejection and DSA-negative, C4d-negative MVI. BK nephropathies were systematically searched using immunohistochemical detection of SV40 large T antigen in renal allograft biopsies and excluded (Fig 1).

### [^18^F]FDG PET/CT imaging

PET/CT was performed with late acquisitions (180 [180–190] minutes) after [^18^F]FDG intravenous injection (222 [193–257] Mbq) within a 24-hour period before or after the surveillance biopsy (0.51 [0.47–1.00] day), prior to any modification of immunosuppressive regimens. The mSUV of kidney cortex was measured and averaged from 4 volumes of interest (VOI) distributed in the upper (n = 2) and lower (n = 2) poles, as previously described^13^. The mSUV of kidney was normalized to the mSUV of the psoas muscle (VOI of 20 ml) as reference tissue [19]. PET and CT images were acquired using center-specific devices: CHU Liège included the cross-calibrated analog GEMINI TF Big Bore and GEMINI TF 16 PET/CT systems (Philips Medical Systems, Cleveland, OH, USA), as well as the digital BIOGRAPH Vision 600 (Siemens); UZA used the digital GE Discovery MI 4R (GE HealthCare). UZB used the cross-calibrated analog BIOGRAPH mCT20 and mCT128 (Siemens).

### Statistics

The results are presented as means and standard deviations (SD) or medians (Q1 - Q3) for quantitative variables and as frequency tables for qualitative variables. The normality of the parameters was tested using the Shapiro-Wilk test. A logarithmic transformation or a square-root transformation was applied in cases where the distribution was skewed. Calculations were carried out on the maximum number of data available and missing values were not replaced. To compare parameters between centers, we used the ANOVA test and Scheffé’s multiple comparisons (means), the Kruskal-Wallis test and Dwass, Steel, Critchlow-Fligner (DSCF) multiple comparisons (medians), and the chi-square test and Bonferroni correction for multiple comparisons (proportions). Furthermore, to take account of possible variations between the centres, the mean (m) and SD of the 3 PET parameters were calculated for each center: renal mSUV_mean_; psoas SUV_mean_; mSUVR and the Z-scores were calculated, using the equation (parameter – m)/ SD. The correlation between the mSUVR and Banff “ti” score was assessed using $R=\frac{\text{cov}(x,\;y)}{\sigma_{x}\;\sigma_{y}}$. To study the risk of SCR (including AMR, TCMR, and MVI) based on mSUVR or Z-score of mSUVR, a stratified logistic regression by center was used and odds ratios (OR) and 95% confidence intervals (95%CI) were reported. Stratified logistic regression models by center were then performed by forcing the SUV value or ratio (or the corresponding Z-scores) and using stepwise selection of parameters with a p-value < 0.10 in univariate analysis. The sample size calculation on the basis of a 7% incidence of SCR [16], with α/β errors of 5% and 80%, respectively, estimated that 156 [^18^F]FDG PET/CT were needed to statistically test its diagnostic performance in biopsy-proven SCR detection. Results were considered significant at a 5% uncertainty level (p < 0.05). Calculations were performed using SAS version 9.4 (SAS Institute Inc., Cary, NC, USA). All relevant data are within the paper and its Supporting Information files.

## Results

From January 2021 to March 2025, we prospectively performed 191 [^18^F]FDG PET/CT in adult KTRs who underwent surveillance transplant biopsy at 91 [87–98] days *post* KTx. Six cases were excluded from the analysis because of BK polyomavirus viremia > 3 log_10_ copies (n = 4) or uninterpretable histology (n = 2) (Fig 1). The mean age of the 185-KTR cohort was 55.0 ± 12.8 years with a sex ratio (M/F) of 1.76 and a mean body mass index (BMI) of 25.7 ± 4.4 kg/m^2^. The mean age of donors was 48.4 ± 12.3 years with a sex ratio of 1.53 and a mean BMI of 25.8 ± 4.6 kg/m^2^ (Table 1). Centers statistically differed by (i) the cold ischemia time (421 [258–747] minutes, p = 0.0005); (ii) the median interval between KTx and transplant biopsy (91 [87–98] days, p = 0.0004); serum creatinine levels (1.31 [1.07–1.54] mg/dL, p = 0.013); and urinary protein/creatinine ratio (119 [81–190] mg/g, p = 0.0001) (S1 Table).

**Fig 2:**
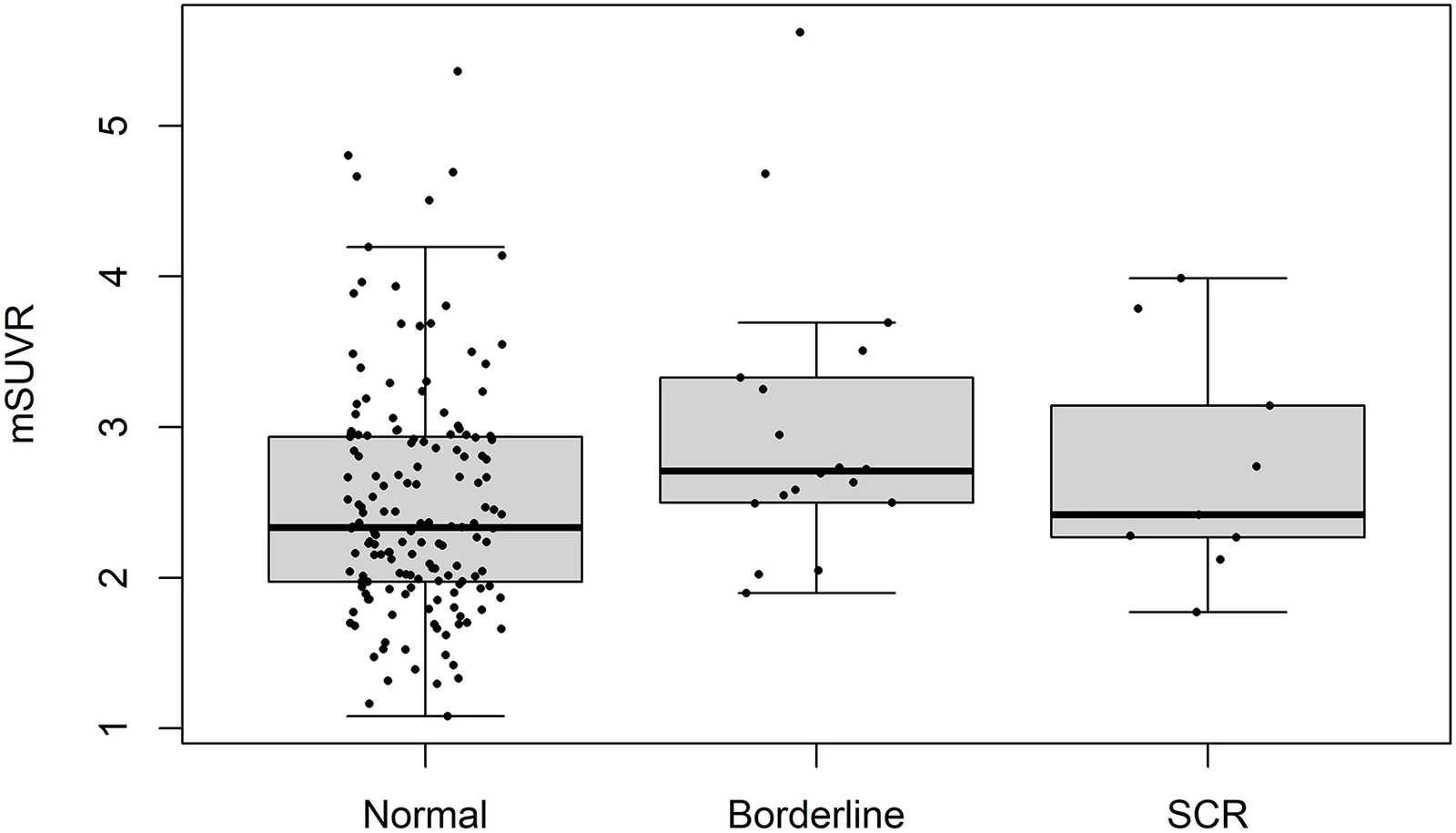
The kidney/psoas ratio of SUV_mean_ (mSUVR) upon histopathological categories. The 185-KTR cohort was categorized upon Banff 2022-based histology: normal (n = 158); borderline (n = 18); subclinical rejection (SCR, n = 9). The mSUVR reached 2.33 [1.97 - 2.93], 2.71 [2.50 −3.33] and 2.42 [2.27 - 3.14] in normal, borderline and SCR groups, respectively.

The 185-KTR cohort was categorized upon Banff 2022-based histology: normal (n = 158); borderline (n = 18); SCR (n = 9, including 6 T-cell-mediated rejection (TCMR-1A) and 3 DSA-negative C4d-negative MVI) (S1 Fig). Immunoreactivity against C4d was detected in 1 case with no features of AMR and no DSA (i.e., “isolated C4d positivity”). No AMR was diagnosed in our surveillance cohort. No significant correlation was observed between the mSUVR and ti score (R = 0.032, p-value = 0.67) or acute composite Banff score (i.e., the sum of g, ptc, t, i, and v; R = 0.045, p-value = 0.54). The mSUVR reached 2.33 [1.97–2.93], 2.71 [2.50–3.33] and 2.42 [2.27–3.14] in normal, borderline and SCR groups, respectively (Fig 2). In univariate analysis stratified by center, the risk of non-normal histology (n = 27, including borderline and SCR) increased with donor age (OR=1.06 [1.01–1.10], p = 0.009), serum creatinine level (OR=6.15 [1.17–32.42], p = 0.032), and mSUVR (OR=5.16 [1.21–22.11], p = 0.027) (S2 Table). A similar finding was observed with the Z-score of the mSUVR: For each increase of 1 standard deviation (i.e., 0.86 for UZA-DIGITAL and UZB-ANALOGIC; 0.51 for CHU-ANALOGIC; and 0.72 for CHU-DIGITAL), the risk of abnormal histology is multiplied by 1.64 (p = 0.014) (S2 Table). In multivariate models stratified by center, mSUVR was not significantly associated with the risk of non-normal histology (OR=4.11 [0.91–18.49], p = 0.07) after adjustment for the age of the donor. The Z-score of mSUVR was significantly associated with the risk of non-normal histology (OR=1.542 [1.021–2.328], p = 0.04), after adjustment for the age of the donor (OR=1.051 [1.008–1.095], p = 0.019) (S3 Table).

Focusing on the risk of biopsy-proven SCR (n = 9), it was not significantly associated with any clinical, biological or radiological parameter, including mSUVR or Z-score of mSUVR, in univariate models stratified by center. The individual mSUVR of the TCMR subgroup were: 1.77, 2.12, 2.27, 2.28, 2.14, and 3.79. The mSUVR of the MVI subgroup were: 2.42, 2.74, and 3.99. Regarding the previously proposed >2.4 mSUVR threshold [16], no significant difference was observed between the proportion of biopsy-proven SCR above the threshold (5/9, 55.6%) and the proportion of normal or borderline histologies above the threshold (87/176, 49.4%) (p = 0.75).

## Discussion

The term “SCR” was introduced by Rush et al. in the 1990’s to refer to stable allografts displaying an interstitial infiltrate and tubulitis [20]. The diagnosis of SCR is clinically relevant, given the efficiency of early treatment of SCR to decrease the incidence of late allograft rejection episodes and the occurrence of fibrosis, thereby improving long-term graft function in KTR [3]. However, the systematic indication of surveillance biopsy remains debatable, and it is not currently part of the KDIGO 2009 guidelines, rather emphasizing broad justifications for per-cause biopsy [21]. The development and validation of alternative non-invasive approaches “ruling out” SCR may help avoid biopsy-associated complications and limitations in stable patients with a low risk of SCR [22]. In a pilot prospective monocentric 92-KTR cohort, we postulated that a ratio of kidney/psoas mSUVR lower than 2.4 would non-invasively exclude SCR, with a negative predictive value of 98% [16]. In the present multicenter validation study, we do not confirm that mSUVR reliably exclude SCR in stable non-sensitized KTR at 3 months *post* KTx. Indeed, the median mSUVR in the group with biopsy-proven SCR, including 6 TCMR and 3 MVI, reached 2.42 [2.27–3.14]. A novel diagnostic threshold for SCR based on mSUVR would be lowered to 2.12, with a sensitivity of 88.9 [51.8–99.7], a specificity of 33.5 [26.6–41.0] and a negative predictive value of 98.3 [91.1–99.9]. The “MVI, DSA-negative, C4d-negative” category was introduced in the Banff 2022 update to reflect its association with adverse graft outcomes, including an increased risk of graft failure [23]. Typically, MVI is a histological feature of immune-mediated injury at the capillary interface of renal allografts, characterized by immune cell infiltration into glomerular and peritubular capillaries. Emerging evidence from transcriptome analyses highlights natural killer cells as possible effectors, regardless of DSA status. No AMR was diagnosed in the present series. The “Borderline” category is a well-known limitation of the Banff classification, with recent controversy about the tubulitis without interstitial inflammation (i.e., “i0 tx”) [24]. In 2009, the “Borderline” definition included foci of tubulitis (t1, t2, or t3) with minor interstitial infiltration (i0 or i1) or interstitial infiltration (i2, i3) with mild (t1) tubulitis. By contrast, the Banff 2019 classification narrowed this definition as follows: tubulitis (t1, 2, or 3) and interstitial inflammation (i1) or tubulitis (t1) and interstitial inflammation (i2 or i3) (https://banfffoundation.org). Molecular microscopy and omics are currently ongoing to better specify this “Borderline” phenotype from both diagnostic and prognostic perspectives [25]. Similarly, one may hypothesize that non-invasive biomarkers and/or imaging-based markers may help better distinguish “borderline/normal” lesions from “borderline/rejection” lesions by reflecting the *in toto* intensity of the inflammation within the kidney allograft. In our cohort, the mSUVR of kidney allograft with “borderline histology” reached 2.71 [2.50–3.33], which was significantly higher than the “normal histology” (2.33 [1.97–2.93], p = 0.022). Recent investigations have highlighted the continuous nature of the rejection process regardless of the underlying disease cause, which questions the dichotomization of the Banff classification [26].

The limitations of our study include the low incidence of pathological cases, although one may argue that a 13% incidence of “Borderline + SCR” cases is consistent with 10–20% range reported from most studies under modern immunosuppression in the early post-transplant course. Still, we have to admit that, despite the small number of cases, [18F]FDGPET/CT cannot reliably rule out SCR, since 5/6 TCMR showed mSUVR below the 2.4 threshold established by our previous study [16]. Given the multicentric design of the present trial, the quantification of [^18^F]FDG uptake in the renal allograft was prospectively performed in each center according to the above-detailed protocol. The use of multiple independent 1-ml VOI distributed in lower and upper renal poles aimed to avert sampling errors and consider a large representative zone of renal parenchyma. The use of the mSUVR referenced to the psoas muscle aimed to further reduce the variability of the radiotracer biodistribution between (i) patients and (ii) PET/CT devices [19,27]. Exams were indeed performed on both analogic and digital [^18^F]FDG PET/CT systems, the latter, based on silicon photomultipliers, providing superior resolution, sensitivity, and improved image quality. Although all PET/CT devices were EARL-certified and analyses were stratified by center to account for inter-scanner, images were not centrally reviewed, and cross-calibration across the four devices was not feasible within the pragmatic, real-world design of this multicenter study, which may have introduced residual quantitative variability in [18F]FDG uptake measurements not fully captured by center-stratified Z-scores. The nature of the radiotracer [^18^F]FDG explains *per se* the poor specificity of [^18^F]FDGPETCT in the differential diagnosis of inflammatory conditions. Radiomics is the high-throughput extraction and analysis of quantitative features from medical images to characterize tissue phenotypes. Radiomics has been increasingly used in inflammatory and infectious diseases, such as sarcoidosis and vasculitis [28,29]. One may hypothesize that radiomics, including (semi-) automatic segmentation methods of the renal cortex, may improve the diagnostic yield of [^18^F]FDG PET/CT in SCR screening. Combining high negative predictive value with high sensitivity would ensure a low false negative rate when considering both the population- and disease-specific performance, and make a more convincing case for avoidance of unnecessary biopsies.

In conclusion, our prospective multicentric cohort including 185 cases combining transplant biopsy and [^18^F]FDG PET/CT at 3 months *post* kidney transplantation did not validate the 2.4 mSUVR threshold to safely exclude SCR. The low SCR incidence, while consistent with contemporary cohorts, limits definitive conclusions regarding the diagnostic yield of [^18^F]FDG PET/CT for SCR [30]. Larger multicenter and adequately powered cohorts are warranted to test the utility of innovative imaging-based markers in the follow-up of KTR.

## Supporting information

S1 Table Characteristics of the multicenter cohort. PET and CT images were acquired using center-specific devices: CHU Liège included cross-calibrated GEMINI TF Big Bore and GEMINI TF 16 PET/CT systems (Philips Medical Systems), as well as BIOGRAPH Vision 600 (Siemens); UZA used the digital GE Discovery MI 4R (GE HealthCare); UZB used cross-calibrated BIOGRAPH mCT20 and mCT128 (Siemens). Data are expressed as mean ± standard deviation; median [interquartile range]. *P-value = ANOVA test (means) or Kruskal-Wallis test (medians) or chi-square (proportions) test; ^abc^: Pairwise Scheffe or Dwass, Steel, Critchlow-Fligner (DSCF) comparisons and chi-square test with Bonferroni’s correction. BMI, body mass index; DBD, donor after brain death; DCD, donor after circulatory death; DSA, donor-specific anti-HLA antibodies; FDG, fluorodeoxyglucose; KTx, kidney transplantation; LD, living donor; MMF, mycophenolate mofetyl; MPA, mycophenolic acid; mTOR, mammalian target of rapamycin; PET/CT, positron-emission tomography/ computed tomography; PRA, panel reactive antigens. SCR, biopsy-proven subclinical acute rejection.
(PDF)

S2 Table Univariate analysis of non-normal *versus* normal histology stratified by center. BMI: body mass index; CS: corticosteroids; DBD; donor after brain death; DCD: donor after circulatory death; DSA: donor-specific antibodies; FDG: fluorodeoxyglucose; KTx: kidney transplantation; IS: immunosuppression; LD: living donor; MMF: mycophenolate mofetyl; MPA: mycophenolic acid; mTOR: mammalian target of rapamycin; PET/CT: positron-emission tomography/ computed tomography; PRA: panel reactive antigens; OR: odds ratio; SE: Parameter estimate; 95%CI: 95% confidence interval.
(PDF)

S3 Table Multivariate analysis of non-normal *versus* normal histology stratified by center. OR, odds ratio, SE, standard error, 95%CI, 95% confidence interval.
(PDF)

S1 Fig Flowchart of the cohort. Between January 2021 and March 2025, 191[^18^F]FDG PET/CT were prospectively performed at the time of surveillance transplant biopsy at ~3 months *post* transplantation in 3 independent centers (i.e., Universitair Ziekenhuis Antwerpen (UZA), Universiteit Ziekenhuis Brussels (UZB), and Centre Hospitalier Universitaire de Liège (CHU Liège)). At CHU Liège, [^18^F]FDGPET/CT images were acquired on 2 different machines. Six cases were excluded (4 PCR-proven BK nephropathies and 2 uninterpretable histology). Biopsies were diagnosed as normal (n = 158), borderline (n = 18) or AR (n = 9, including 6 T-cell-mediated rejection (TCMR) and 3 microvascular inflammation (MVI)).
(PDF)
