# Supplementary material for: A multicenter prospective validation cohort does not support the use of kidney/psoas [18F]FDG uptake in the diagnosis of kidney allograft subclinical rejection
Source: PLoS One. 2026 Sep 2;21(9):e0345426. doi: 10.1371/journal.pone.0345426 (PMC13537687; doi:10.1371/journal.pone.0345426)
Supplement: Supplementary file 1 [file pone.0345426.s001.pdf]

Supplementary Table 1. Characteristics of the multicenter cohort

	Global<br>N = 185	UZA-DIGITAL<br>N = 42	UZB-ANALOGIC<br>N = 62	CHU-ANALOGIC<br>N = 24	CHU-DIGITAL<br>N = 57	P-value*
**RECIPIENT AT BIOPSY**						
Age (years), mean ± SD	55.0 ± 12.8	53.3 ± 14.5	53.1 ± 12.8	57.3 ± 13.2	57.3 ± 11.1	0.19
BMI (kg/m²), mean ± SD	25.7 ± 4.35	26.0 ± 4.70	26.5 ± 4.10	24.0 ± 3.90	25.4 ± 4.40	0.12
Sex (Female)	67 (36.2)	14 (33.3)	24 (38.7)	9 (37.5)	20 (35.1)	0.95
PRA max						0.43
<5%	140 (76.9)	29 (74.4)	46 (74.2)	17 (70.8)	48 (84.2)	
5-85%	30 (16.5)	8 (20.5)	9 (14.5)	5 (20.8)	8 (14.0)	
>85%	12 (6.6)	2 (5.1)	7 (11.3)	2 (8.3)	1 (1.8)	
**DONOR**						
Age (years), mean ± SD	48.4 ± 12.3	52.5 ± 11.4	47.0 ± 12.0	44.5 ± 15.1	48.5 ± 11.4	0.046
BMI (kg/m²), mean ± SD	25.8 ± 4.62	25.6 ± 3.80	25.3 ± 4.59	26.0 ± 3.11	26.4 ± 5.65	0.55
Sex (Female (%))	73 (39.7)	16 (38.1)	27 (44.3)	7 (29.2)	23 (40.4)	0.64
Type						0.013
DBD	95 (51.4)	17 (40.5)	35 (56.5)	14 (58.3)	29 (50.9)	
DCD	62 (33.5)	11 (26.2)	19 (30.6)	9 (37.5)	23 (40.4)	
LD	28 (15.1)	14 (33.3)	8 (12.9)	1 (4.2)	5 (8.8)	
**TRANSPLANTATION**						
Cold ischemia time (min), median [Q1-Q3]	421 [258 - 747]	334 [47.0 - 539] [ab]	576 [364 - 772] [ac]	495 [288 - 850] [b]	356 [255 - 546] [c]	0.0005
Delayed graft function (%)	25 (13.5)	5 (11.9)	12 (19.4)	5 (20.8)	3 (5.3)	0.097
**STATUS AT TIME OF BIOPSY**						
Delay between Tx and biopsy (days), median [Q1-Q3]	91.0 [87.0 - 98.0]	88.0 [83.0 - 91.0] [ab]	93.5 [88.0 - 100] [a]	90.5 [84.5 - 109]	92.0 [90.0 - 98.0] [b]	0.0004
Calcineurin Inhibitors (Yes, %)			[a]		[a]	0.0005
Tacrolimus	174 (94.1)	41 (97.6)	52 (83.9)	24 (100.0	57 (100.0	
CsA	11 (5.9)	1 (2.4)	10 (16.1)	0 (0.0)	0 (0.0)	
Anti-metabolites (Yes, %)		[a]	[a]			0.0021
None	7 (3.8)	6 (14.3)	0 (0.0)	1 (4.2)	0 (0.0)	

	Global N = 185	UZA-DIGITAL N = 42	UZB-ANALOGIC N = 62	CHU-ANALOGIC N = 24	CHU-DIGITAL N = 57	P-value*
MMF	174 (94.1)	36 (85.7)	59 (95.2)	23 (95.8)	56 (98.2)	
AZA	4 (2.2)	0 (0.0)	3 (4.8)	0 (0.0)	1 (1.8)	
mTOR inhibitors (Yes (%))	6 (3.2)	4 (9.5)	2 (3.2)	0 (0.0)	0 (0.0)	0.046
Corticosteroids (Yes (%))	181 (97.8)	39 (92.9)	61 (98.4)	24 (100.0	57 (100.0	0.078
Creatinine (mg/dL), median [Q1-Q3]	1.31 [1.07 - 1.54]	1.43 [1.19 - 1.58]	[a] 1.23 [1.01 - 1.46]	[a] 1.23 [1.02 - 1.49]	1.36 [1.18 - 1.56]	0.013
Proteinuria (mg/g creat) , median [Q1-Q3]	119 [81.5 - 190]	180 [125 - 246]	[abc] 100 [60.0 - 132]	[a] 108 [80.2 - 169]	[b] 119 [85.1 - 201]	[c] <.0001
DSA						0.38
None	177 (96.2)	40 (95.2)	57 (91.9)	23 (100.0	57 (100.0	
HLA Class I	3 (1.6)	1 (2.4)	2 (3.2)	0 (0.0)	0 (0.0)	
HLA Class II	4 (2.2)	1 (2.4)	3 (4.8)	0 (0.0)	0 (0.0)	
**PET-CT**						
Blood glucose (mg/dL), median [Q1-Q3]	104 [93.0 - 120]	101 [94.0 - 122]	100 [92.0 - 116]	108 [96.0 - 125]	105 [95.0 - 124]	0.25
FDG activity (mBq), median [Q1-Q3]	222 [193 - 257]	227 [193 - 267]	[ab] 260 [226 - 296]	[acd] 220 [216 - 227]	[de] 186 [157 - 214]	[bce] <.0001
Delay injection-PET/CT (min), median [Q1-Q3]	180 [180 - 190]	183 [180 - 186]	[abc] 180 [176 - 180]	[ade] 194 [181 - 199]	[ce] 189 [181 - 194]	[bd] <.0001
Delay between biopsy and PET-CT/CT (days), Median [Q1-Q3]	0.51 [0.00 - 1.00]	-1.0 [-1.00 - -1.00]	[abc] 1.00 [1.00 - 1.00]	[acd] 0.52 [0.52 - 0.52]	[bce] 0.51 [0.51 - 0.51]	[cde] <.0001
**SUV**						
mSUV, median [Q1 – Q3]	1.86 [1.68 - 2.16]	1.92 [1.76 - 2.19]	[ab] 2.09 [1.78 - 2.24]	[cd] 1.69 [1.53 - 1.92]	[ac] 1.73 [1.63 - 1.91]	[bd] <.0001
mSUVR, median [Q1 – Q3]	2.37 [2.01 - 2.95]	2.57 [2.05 - 2.93]	2.55 [2.06 - 3.15]	[a] 2.06 [1.85 - 2.36]	[a] 2.36 [1.97 - 2.86]	0.031
**Banff 2023 Classification**						
Normal (Yes (%))	158 (85.4)	31 (73.8)	52 (83.9)	22 (91.7)	53 (93.0)	0.070
Borderline (Yes (%))	18 (9.7)	9 (21.4)	5 (8.1)	1 (4.2)	3 (5.3)	
SCR (Yes (%))	9 (4.9)	2 (4.8)	5 (8.1)	1 (4.2)	1 (1.8)	
