# Supplementary material for: A multicenter prospective validation cohort does not support the use of kidney/psoas [18F]FDG uptake in the diagnosis of kidney allograft subclinical rejection
Source: PLoS One. 2026 Sep 2;21(9):e0345426. doi: 10.1371/journal.pone.0345426 (PMC13537687; doi:10.1371/journal.pone.0345426)
Supplement: Supplementary file 2 [file pone.0345426.s002.pdf]

Supplementary Table 2. Univariate analysis of non-normal *versus* normal histology stratified by center

	N	SE	OR	95% CI OR		P-value
**RECIPIENT AT BIOPSY**						
Age (years)	185	0.0052 (0.016)	1.005	0.974	1.038	0.75
BMI (kg/m²)	185	0.068 (0.049)	1.070	0.972	1.177	0.17
Sex M vs. F	185	0.55 (0.47)	1.742	0.689	4.405	0.24
PRA max 5-85% vs. <5%	182	0.11 (0.55)	1.119	0.378	3.315	0.76
>85% vs. < 5%		-0.75 (1.08)	0.473	0.057	3.922	
**DONOR**						
Age (years)	185	0.056 (0.021)	1.057	1.014	1.102	**0.009**
BMI (kg/m²)	185	0.018 (0.048)	1.018	0.928	1.118	0.70
Sex M vs. F	184	0.15 (0.44)	1.166	0.495	2.745	0.73
Type DCD vs. DBD	185	0.68 (0.46)	1.979	0.797	4.914	0.24
LD vs. DBD		-0.18 (0.65)	0.832	0.231	3.003	
**TRANSPLANTATION**						
Tx Cold ischemia time (min) (squareroot)	183	0.018 (0.030)	1.018	0.960	1.080	0.55
Early graft function Delayed vs. Immediate	185	0.38 (0.56)	1.469	0.487	4.430	0.50
**STATUS AT TIME OF BIOPSY**						
Delay between Tx and biopsy (days) (Log)	184	0.87 (1.31)	2.393	0.183	31.309	0.51
IS CNI CSA vs. Tacrolimus	185	0.091 (0.84)	1.095	0.210	5.726	0.91
IS MMF MMF vs. None	185	-0.30 (0.91)	0.742	0.125	4.392	0.95
AZA vs. None		-14.6 (1560)	<0.001	<0.001	>999	
IS MTOR inhibitor Yes. vs. No	185	-0.42 (1.13)	0.658	0.072	6.005	0.71
IS CS Yes vs. No	185	-1.24 (1.03)	0.288	0.038	2.181	0.23
Creatinine (mg/dL) (Log)	185	1.82 (0.85)	6.147	1.165	32.418	0.03
Proteinuria (mg/g creat) (Log)	185	-0.32 (0.35)	0.725	0.363	1.448	0.36
DSA Class I vs. None	184	2.22 (1.25)	9.198	0.789	107.204	0.20
Class II vs. None		0.50 (1.19)	1.641	0.161	16.753	
**PET-CT**						
Blood glucose (mg/dL) (Log)	182	0.47 (1.06)	1.594	0.199	12.793	0.66
FDG activity (mBq)	185	0.0046 (0.0045)	1.005	0.996	1.013	0.30
Delay injection-PET (min) (Log)	185	3.38 (5.00)	39.775	0.002	>999	0.46
Delay between biopsy and PET-CT (days) (Log)	138	0.36 (0.27)	1.438	0.850	2.433	0.18
**SUV**						
mSUV (Log)	185	1.90 (1.17)	6.693	0.670	66.877	0.11
mSUVR (Log)	185	1.64 (0.74)	5.161	1.205	22.106	**0.027**
**Z-scores of SUV**						
Z-mSUV	185	0.30 (0.20)	1.335	0.907	1.964	0.14
Z-mSUVR	185	0.49 (0.20)	1.639	1.105	2.433	**0.014**
