# Supplementary material for: A multicenter prospective validation cohort does not support the use of kidney/psoas [18F]FDG uptake in the diagnosis of kidney allograft subclinical rejection
Source: PLoS One. 2026 Sep 2;21(9):e0345426. doi: 10.1371/journal.pone.0345426 (PMC13537687; doi:10.1371/journal.pone.0345426)
Supplement: Supplementary file 3 [file pone.0345426.s003.pdf]

Supplementary Table 3. Multivariate analysis of non-normal *versus* normal histology stratified by center

Stepwise selection models with PET-CT parameter forced in the model	N	SE	OR	95% CI OR		p-value
Kidney/Muscle Mean SUVMean (Log)		1.41 (0.77)	4.108	0.913	18.485	0.066
Age of the donor (years)		0.050 (0.021)	1.051	1.009	1.096	**0.018**
	185					
Z-score mSUVR		0.43 (0.21)	1.542	1.021	2.328	**0.040**
Age of the donor (years)		0.0496 (0.021)	1.051	1.008	1.095	**0.019**

OR: odds ratio, SE: parameter estimate; 95%CI: 95% confidence interval.
