# Supplementary material for: A multicenter prospective validation cohort does not support the use of kidney/psoas [18F]FDG uptake in the diagnosis of kidney allograft subclinical rejection
Source: PLoS One. 2026 Sep 2;21(9):e0345426. doi: 10.1371/journal.pone.0345426 (PMC13537687; doi:10.1371/journal.pone.0345426)
Supplement: Supplementary file 4 [file pone.0345426.s004.pdf]

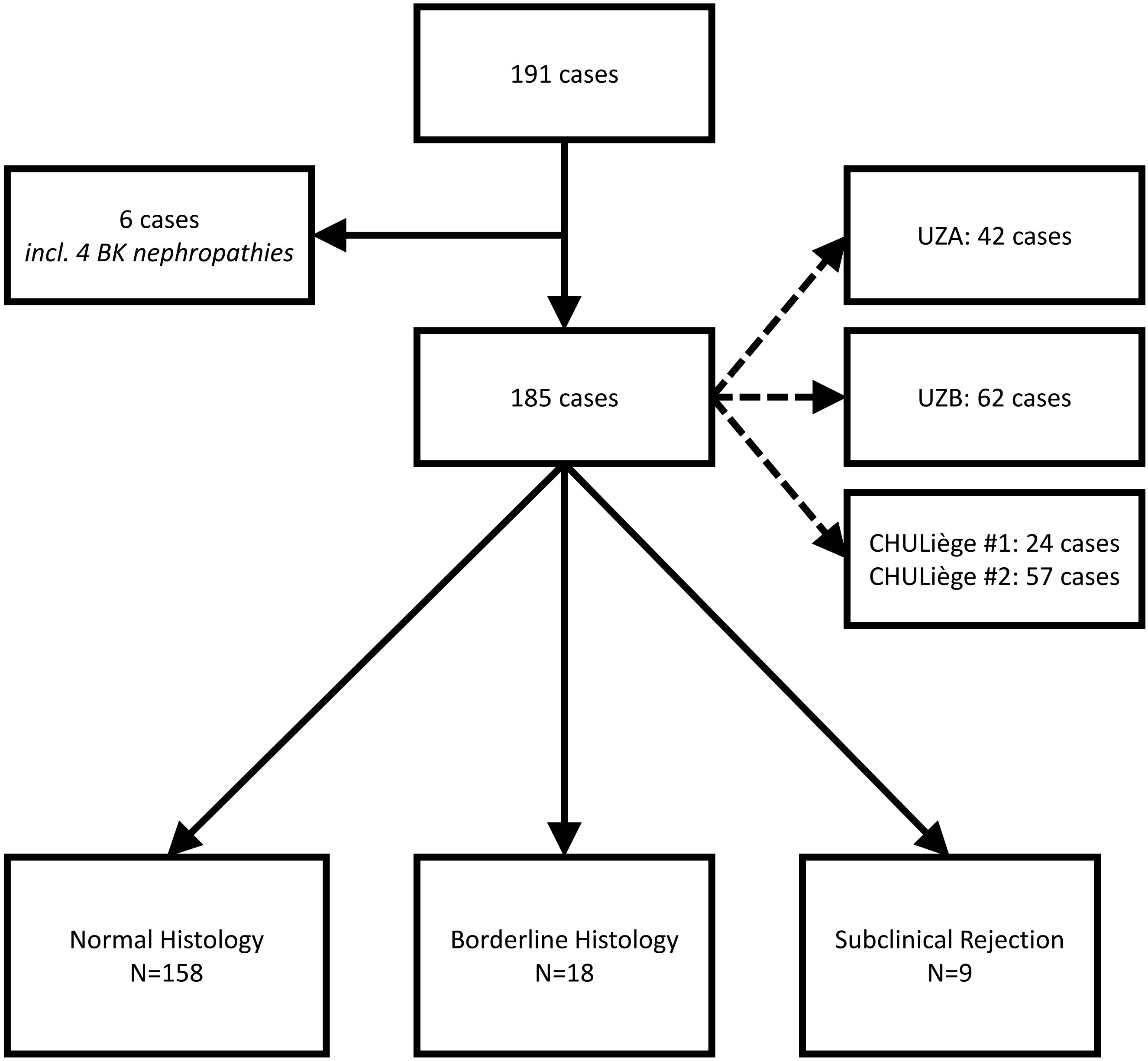
191 cases
6 cases
incl. 4 BK nephropathies
185 cases
UZA: 42 cases
UZB: 62 cases
CHULiège #1: 24 cases
CHULiège #2: 57 cases
Normal Histology
N=158
Borderline Histology
N=18
Subclinical Rejection
N=9
