# Supplementary material for: A multicenter prospective validation cohort does not support the use of kidney/psoas [18F]FDG uptake in the diagnosis of kidney allograft subclinical rejection
Source: PLoS One. 2026 Sep 2;21(9):e0345426. doi: 10.1371/journal.pone.0345426 (PMC13537687; doi:10.1371/journal.pone.0345426)
Supplement: Supplementary file 5 [file pone.0345426.s006.docx]

PONE-D-26-10474

A multicenter prospective validation cohort does not confirm the diagnostic yield of [18F]FDG PET/CT imaging in kidney allograft subclinical rejection

PLOS One

Dear Dr. Jouret,

Thank you for submitting your manuscript to PLOS ONE. After careful consideration, we feel that it has merit but does not fully meet PLOS ONE’s publication criteria as it currently stands. Therefore, we invite you to submit a revised version of the manuscript that addresses the points raised during the review process.

==============================
**ACADEMIC EDITOR:**

**Main concerns:** please specify better what makes your paper different from similar research available currently in the literature, and why your results are somehow in contrast with published evidence

We would like to thank the Academic Editor for emphasizing that our prospective multicenter cohort study has merit and inviting us to submit a revised version of the manuscript including all suggestions and comments of Reviewers #1 and #2. Especially, we have better discussed what makes our paper different from similar research available currently in the literature, and why our results are somehow in contrast with published evidence. The pilot study (PMID: 31841263) included only 6 cases of subclinical rejection, which may have led to an overestimation of diagnostic performance and instability of the proposed 2.4 mSUVR threshold. Furthermore, this pilot study was monocentric. Focusing on the 158 cases with biopsy-proven normal histology of the present multicenter cohort, the inter-center variability of the Kidney SUV_mean_ was greater than intra-center variability of the Kidney SUV_mean_ (0.91 vs. 0.14, p=0.0003). By contrast, the inter-center variability of the Psoas SUV_mean_ was similar to the intra-center variability of the Psoas SUV_mean_ (0.055 vs. 0.045, p=0.31). This prompted us to use the kidney/psoas mSUVRatio as an attempt of intra-individual “normalization”. Still, the multicenter design inherently introduced heterogeneity (e.g. different PET/CT devices, analog vs digital technologies, acquisition and reconstruction protocols), which may affect quantitative FDG uptake measurements. Following the advice of “harmonization” of Reviewer #2, we have calculated the Z-scores in our multicenter cohort. For each center, we have calculated the mean (m) and standard deviation (SD) of the 3 PET parameters: renal mSUV_mean_; psoas SUV_mean_; mSUVR. For each parameter in the center, we have calculated Z, using the equation (parameter – m) / SD. The Z-score is a standardized statistical measure, i.e. the mean and standard deviation of Z are 0 and 1. It quantifies the number of standard deviations by which an observation differs from the mean of a distribution.

In univariate analysis, the risk of non-normal histology (n=27, including Borderline and SCR) increased with the Z-score of the mSUVR: For each increase of 1 standard deviation (i.e. 0.86 for UZA-DIGITAL and UZB-ANALOGIC; 0.51 for CHU-ANALOGIC; and 0.72 for CHU-DIGITAL), the risk of abnormal histology is multiplied by 1.64 (p=0.014) (**Supplementary Table 2**). In multivariate models, the Z-score of mSUVR was significantly associated with the risk of non-normal histology (OR=1.542 [1.021–2.328, p=0.04). The [18F]-FDG uptake reflects metabolic activity of inflammatory cells. Subclinical rejection, by definition, may involve limited or focal inflammation, potentially below the detection threshold of PET/CT imaging. Indeed, our multicenter cohort includes a broader spectrum of lesions, including the classical T-cell–mediated rejection and the recently Banff-recognized entity “microvascular inflammation (MVI) without DSA or C4d”. These lesions may involve different inflammatory patterns. As a whole, the monocentric studies (PMID: 31841263; PMID: 26302136; PMID: 34191101) may reflect a more homogeneous and selected population (distinguishing per-protocol *versus* per-cause biopsies, as an example), whereas the present multicentric cohort better reflects real-world heterogeneity, leading to reduced diagnostic performance of [18F]-FDG PET/CT imaging.

**Minor concerns:** please analyze additional PET-derived metrics as MTV or TLG; please specify better whether harmonization procedures were applied to overcome the drawbacks from obtaining functional measures from different scanners.

We would like to thank the Academic Editor and Reviewer #2 for helping us to strengthen the harmonization process of our multicenter data. Image acquisition and reconstruction were pragmatically done in real-life settings. Cross-calibration between the different PET/CT devices was not feasible for the specific purpose of the present study. Still, all PET/CT devices are routinely calibrated and EARL-certified. The quantification of renal [18F]-FDG uptake was performed manually following a standardized protocol. We have previously showed that the intra-class correlation coefficient ICC for inter-observer reproducibility for each VOI was 0.87 [0.81-0.91], 0.87 [0.81-0.91], 0.85 [0.78-0.89] and 0.83 [0.76-0.88] for the upper to the lower renal poles, respectively (PMID: 32165653). Furthermore, renal [18F]-FDG uptake was individually normalized to [18F]-FDG uptake by the psoas muscle of the patient. Finally, following the suggestions of Reviewer #2, we have calculated the z-scores in our multicenter cohort (see supra). Additional PET-derived metrics, such as metabolic tumor volume (MTV), would be pertinent but require multidimensional procedures of analysis, including automated segmentation of the renal graft standardized for the 4 PET/CT devices used in the present multicenter study. These highly relevant PET-derived metrics require further investigations, as detailed in the Discussion.

On the basis of the comments and suggestions of the Academic Editor and the Reviewers, we propose to scientifically nuance the title of the manuscript focusing on the mSUVR, as follows: “A multicenter prospective validation cohort does not support the use of kidney/psoas [18F]FDG uptake in the diagnosis of kidney allograft subclinical rejection”.

==============================

Please submit your revised manuscript by May 29 2026 11:59PM. If you will need more time than this to complete your revisions, please reply to this message or contact the journal office at [plosone@plos.org](mailto:plosone@plos.org). When you're ready to submit your revision, log on to https://www.editorialmanager.com/pone/ and select the 'Submissions Needing Revision' folder to locate your manuscript file.

Please include the following items when submitting your revised manuscript:

- A letter that responds to each point raised by the academic editor and reviewer(s). You should upload this letter as a separate file labeled 'Response to Reviewers'.
- A marked-up copy of your manuscript that highlights changes made to the original version. You should upload this as a separate file labeled 'Revised Manuscript with Track Changes'.
- An unmarked version of your revised paper without tracked changes. You should upload this as a separate file labeled 'Manuscript'.

If you would like to make changes to your financial disclosure, please include your updated statement in your cover letter. Guidelines for resubmitting your figure files are available below the reviewer comments at the end of this letter.

If applicable, we recommend that you deposit your laboratory protocols in protocols.io to enhance the reproducibility of your results. Protocols.io assigns your protocol its own identifier (DOI) so that it can be cited independently in the future. For instructions see: [https://journals.plos.org/plosone/s/submission-guidelines#loc-laboratory-protocols](https://track.editorialmanager.com/CL0/https:%2F%2Fjournals.plos.org%2Fplosone%2Fs%2Fsubmission-guidelines%23loc-laboratory-protocols/1/010f019d8a28ac09-5d30fefd-7a74-4f3b-a178-85ea92d71cc3-000000/0mK8ymua1tJuBLaPtq94E4Ip4Pz4B29RJ9-TpVc8JT0=258). Additionally, PLOS ONE offers an option for publishing peer-reviewed Lab Protocol articles, which describe protocols hosted on protocols.io. Read more information on sharing protocols at [https://plos.org/protocols?utm_medium=editorial-email&utm_source=authorletters&utm_campaign=protocols](https://track.editorialmanager.com/CL0/https:%2F%2Fplos.org%2Fprotocols%3Futm_medium=editorial-email%26utm_source=authorletters%26utm_campaign=protocols/1/010f019d8a28ac09-5d30fefd-7a74-4f3b-a178-85ea92d71cc3-000000/5bgMaucDX4pRDC7iILDvwmfShJGfoEj5ycnXHf35f5w=258).

As the corresponding author, your ORCID iD is verified in the submission system and will appear in the published article. PLOS supports the use of ORCID, and we encourage all coauthors to register for an ORCID iD and use it as well. Please encourage your coauthors to verify their ORCID iD within the submission system before final acceptance, as unverified ORCID iDs will not appear in the published article. *Only* the individual author can complete the verification step; PLOS staff *cannot* verify ORCID iDs on behalf of authors.

We look forward to receiving your revised manuscript.

Kind regards,

Carmelo Caldarella, Ph.D., M.D.
Academic Editor
PLOS One

Journal Requirements:

When submitting your revision, we need you to address these additional requirements.
1. Please ensure that your manuscript meets PLOS ONE's style requirements, including those for file naming. The PLOS ONE style templates can be found at
[https://journals.plos.org/plosone/s/file?id=wjVg/PLOSOne_formatting_sample_main_body.pdf](https://track.editorialmanager.com/CL0/https:%2F%2Fjournals.plos.org%2Fplosone%2Fs%2Ffile%3Fid=wjVg%2FPLOSOne_formatting_sample_main_body.pdf/1/010f019d8a28ac09-5d30fefd-7a74-4f3b-a178-85ea92d71cc3-000000/QSIT3gDqQ0drtsf3KmVqW4iujMQAzc4ojs0qDjPFB8Q=258) and [https://journals.plos.org/plosone/s/file?id=ba62/PLOSOne_formatting_sample_title_authors_affiliations.pdf](https://track.editorialmanager.com/CL0/https:%2F%2Fjournals.plos.org%2Fplosone%2Fs%2Ffile%3Fid=ba62%2FPLOSOne_formatting_sample_title_authors_affiliations.pdf/1/010f019d8a28ac09-5d30fefd-7a74-4f3b-a178-85ea92d71cc3-000000/EznLl0Pk_u-JUYlswlAJS7H4pHldwndLn2wA91DdL6c=258)

2. We note that you have indicated that there are restrictions to data sharing for this study. For studies involving human research participant data or other sensitive data, we encourage authors to share de-identified or anonymized data. However, when data cannot be publicly shared for ethical reasons, we allow authors to make their data sets available upon request. For information on unacceptable data access restrictions, please see [http://journals](http://journals/).plos.org/plosone/s/data-availability#loc-unacceptable-data-access-restrictions.

Before we proceed with your manuscript, please address the following prompts:

a) If there are ethical or legal restrictions on sharing a de-identified data set, please explain them in detail (e.g., data contain potentially identifying or sensitive patient information, data are owned by a third-party organization, etc.) and who has imposed them (e.g., a Research Ethics Committee or Institutional Review Board, etc.). Please also provide contact information for a data access committee, ethics committee, or other institutional body to which data requests may be sent.

b) If there are no restrictions, please upload the minimal anonymized data set necessary to replicate your study findings to a stable, public repository and provide us with the relevant URLs, DOIs, or accession numbers. Please see <http://www.bmj.com/content/340/bmj.c181.long> for guidelines on how to de-identify and prepare clinical data for publication. For a list of recommended repositories, please see [https://journals](https://journals/).plos.org/plosone/s/recommended-repositories. You also have the option of uploading the data as Supporting Information files, but we would recommend depositing data directly to a data repository if possible.

Please update your Data Availability statement in the submission form accordingly.

3. Thank you for stating the following in the Acknowledgments Section of your manuscript:
The authors cordially thank the patients-participants, the surgeons (A. De Roover, O. Detry, N. Gilbo, N. Meurisse, and M. Vandermeulen), the physicians (L. Vanovermeire and P. Xhignesse), and the members of the local transplant coordination center (J Mornard, F. Sinte and A Warmoes) for their commitment to kidney transplantation at the University of Liege Hospital in Liege, Belgium; the surgeons (T. Chapelle, G. Roeyen, B. Bracke, V. Hartman, B. Hendrikx, E. Liekens), the physicians (M. Dirix, H. de Fijter, V. de Meier), the study coordinator (P. Lievens) and transplant coordinators (G. Van Beeumen, P. Hollants, A. Van de Weyer, L. Van den Bergh, M. De Deyne) at Universitair Ziekenhuis Antwerpen in Antwerp, Belgium; the surgeons, the physicians, the study coordinator and the transplant coordinators at Universitair Ziekenhuis Brussel in Brussels, Belgium. AB is a Fellow of the Fonds National de la Recherche Scientifique. FJ and RH received support from the University of Liège (Fonds Spéciaux à la Recherche; Fondation Léon Fredericq), as well as from ULiège Academic Hospital (Fonds d’Investissement pour la Recherche Scientifique, FIRS). This study was supported by the Belgian Transplant Society.

We note that you have provided funding information that is not currently declared in your Funding Statement. However, funding information should not appear in the Acknowledgments section or other areas of your manuscript. We will only publish funding information present in the Funding Statement section of the online submission form.
Please remove any funding-related text from the manuscript and let us know how you would like to update your Funding Statement. Currently, your Funding Statement reads as follows:
FJ and RH received support from the University of Liège (Fonds Spéciaux à la Recherche; Fondation Léon Fredericq), as well as from ULiège Academic Hospital (Fonds d’Investissement pour la Recherche Scientifique, FIRS). This study was supported by the Belgian Transplant Society.

Please include your amended statements within your cover letter; we will change the online submission form on your behalf.

4. Please include captions for your Supporting Information files at the end of your manuscript, and update any in-text citations to match accordingly. Please see our Supporting Information guidelines for more information: [http://journals](http://journals/).plos.org/plosone/s/supporting-information.

5. If the reviewer comments include a recommendation to cite specific previously published works, please review and evaluate these publications to determine whether they are relevant and should be cited. There is no requirement to cite these works unless the editor has indicated otherwise. 


Reviewers' comments:

Reviewer's Responses to Questions

**Comments to the Author**

1. Is the manuscript technically sound, and do the data support the conclusions?

The manuscript must describe a technically sound piece of scientific research with data that supports the conclusions. Experiments must have been conducted rigorously, with appropriate controls, replication, and sample sizes. The conclusions must be drawn appropriately based on the data presented.

Reviewer #1: Partly

Reviewer #2: Yes

2. Has the statistical analysis been performed appropriately and rigorously?

Reviewer #1: No

Reviewer #2: Yes

3. Have the authors made all data underlying the findings in their manuscript fully available?

The [PLOS Data policy](http://track.editorialmanager.com/CL0/http:%2F%2Fwww.plosone.org%2Fstatic%2Fpolicies.action%23sharing/1/010f019d8a28ac09-5d30fefd-7a74-4f3b-a178-85ea92d71cc3-000000/XvnoSTpi71DXElpYY8JuM4EYuzNssqmDaXdoUJIXROw=258) requires authors to make all data underlying the findings described in their manuscript fully available without restriction, with rare exception (please refer to the Data Availability Statement in the manuscript PDF file). The data should be provided as part of the manuscript or its supporting information, or deposited to a public repository. For example, in addition to summary statistics, the data points behind means, medians and variance measures should be available. If there are restrictions on publicly sharing data—e.g. participant privacy or use of data from a third party—those must be specified.

Reviewer #1: No

Reviewer #2: Yes

4. Is the manuscript presented in an intelligible fashion and written in standard English?

PLOS ONE does not copyedit accepted manuscripts, so the language in submitted articles must be clear, correct, and unambiguous. Any typographical or grammatical errors should be corrected at revision, so please note any specific errors here.

Reviewer #1: No

Reviewer #2: Yes

5. Review Comments to the Author

Please use the space provided to explain your answers to the questions above. You may also include additional comments for the author, including concerns about dual publication, research ethics, or publication ethics. (Please upload your review as an attachment if it exceeds 20,000 characters)

**Reviewer #1:** The study addressed an important topic; however, several areas required revision to meet publication standards.

We would like to thank Reviewer #1 for emphasizing that our study addresses an important topic.

Methodological transparency should be improved, particularly regarding participant selection, experimental procedures, and controls.

The methodology, particularly regarding participant selection, experimental procedures, and controls, has been further detailed in the “Patients and Methods” section of the revised version of the manuscript.

Statistical reporting should be more comprehensive and aligned with standard guidelines.

The statistical methods have been more detailed in the Methods section of the revised manuscript.

The discussion should better integrate existing literature and clearly acknowledge study limitations, including potential bias and generalizability concerns.

We have better discussed what makes our paper different from similar research available currently in the literature, and why our results are somehow in contrast with published evidence. The pilot study (PMID: 31841263) included only 6 cases of subclinical rejection, which may have led to an overestimation of diagnostic performance and instability of the proposed 2.4 mSUVR threshold. Furthermore, this pilot study was monocentric. Focusing on the 158 cases with biopsy-proven normal histology of the present multicenter cohort, the inter-center variability of the Kidney SUV_mean_ was greater than intra-center variability of the Kidney SUV_mean_ (0.91 vs. 0.14, p=0.0003). By contrast, the inter-center variability of the Psoas SUV_mean_ was similar to the intra-center variability of the Psoas SUV_mean_ (0.055 vs. 0.045, p=0.31). This prompted us to use the kidney/psoas mSUVRatio as an attempt of intra-individual “normalization”. Still, the multicenter design inherently introduced heterogeneity (e.g. different PET/CT devices, analog vs digital technologies, acquisition and reconstruction protocols), which may affect quantitative FDG uptake measurements. Following the advice of “harmonization” of Reviewer #2, we have calculated the Z-scores in our multicenter cohort. For each center, we have calculated the mean (m) and standard deviation (SD) of the 3 PET parameters: renal mSUV_mean_; psoas SUV_mean_; mSUVR. For each parameter in the center, we have calculated Z, using the equation (parameter – m) / SD. The Z-score is a standardized statistical measure, i.e. the mean and standard deviation of Z are 0 and 1. It quantifies the number of standard deviations by which an observation differs from the mean of a distribution.

In univariate analysis, the risk of non-normal histology (n=27, including Borderline and SCR) increased with the Z-score of the mSUVR: For each increase of 1 standard deviation (i.e. 0.86 for UZA-DIGITAL and UZB-ANALOGIC; 0.51 for CHU-ANALOGIC; and 0.72 for CHU-DIGITAL), the risk of abnormal histology is multiplied by 1.64 (p=0.014) (**Supplementary Table 2**). In multivariate models, the Z-score of mSUVR was significantly associated with the risk of non-normal histology (OR=1.542 [1.021–2.328, p=0.04), only after adjustment for the age of the donor (OR= 1.051 [1.008–1.095], p=0.019). The [18F]-FDG uptake reflects metabolic activity of inflammatory cells. Subclinical rejection, by definition, may involve limited or focal inflammation, potentially below the detection threshold of PET/CT imaging. Indeed, our multicenter cohort includes a broader spectrum of lesions, including the classical T-cell–mediated rejection and the recently Banff-recognized entity “microvascular inflammation (MVI) without DSA or C4d”. These lesions may involve different inflammatory patterns. As a whole, the monocentric studies (PMID: 31841263; PMID: 26302136; PMID: 34191101) may reflect a more homogeneous and selected population (distinguishing per-protocol *versus* per-cause biopsies, as an example), whereas the present multicentric cohort better reflects real-world heterogeneity, leading to reduced diagnostic performance of [18F]-FDG PET/CT imaging.

Figures and tables should be refined with clearer legends and consistent formatting.

We thank Reviewer #1 for referring to the standardized formatting of Figures and Legends according to PLOS One instructions.

Ethical considerations and approvals, if applicable, should be explicitly stated.

This study has been approved by the Institutional Review Board (IRB) of CHULiège under the number B707202042977, and endorsed by the IRB of UZA and UZB. All these documents have been scanned and downloaded on the PLOS One submission website. Kidney transplantations were performed in accordance with the Declaration of Istanbul.

Overall, substantial revision was required to enhance clarity, reproducibility, and scientific STANDARD

We would like to thank Reviewer #1 for helping us to improve our manuscript according to the scientific standards and instructions of PLOS One.

**Reviewer #2:** In this multicenter prospective investigation, the authors aimed to assess the diagnostic reliability of [18F]FDG PET/CT as a non‑invasive screening modality for the exclusion of subclinical acute kidney allograft rejection (SCR). This study resembles a previous article published in 2019, in which most of the current authors demonstrated the usefulness of FDG PET/CT for excluding SCR in a cohort of 92 patients. However, after applying the same PET‑derived parameter—the mean SUV ratio between the renal cortex and the psoas muscle (mSUVR)—the present study found no significant correlation with the t‑i histological score, nor was mSUVR associated with an increased risk of non‑normal histology in multivariate analysis. The authors therefore conclude that this metric, tested in a larger cohort of 185 patients, is not effective in distinguishing normal, borderline, and SCR cases, and does not provide sufficient diagnostic yield to rule out SCR in clinically stable recipients at 3 months after kidney transplantation.

We would like to thank Reviewer #2 for his/her constructive suggestions and comments about our prospective multicenter cohort study.

We have better discussed what makes our paper different from similar research available currently in the literature, and why our results are somehow in contrast with published evidence. The pilot study (PMID: 31841263) included only 6 cases of subclinical rejection, which may have led to an overestimation of diagnostic performance and instability of the proposed 2.4 mSUVR threshold. Furthermore, this pilot study was monocentric. Focusing on the 158 cases with biopsy-proven normal histology of the present multicenter cohort, the inter-center variability of the Kidney SUV_mean_ was greater than intra-center variability of the Kidney SUV_mean_ (0.91 vs. 0.14, p=0.0003). By contrast, the inter-center variability of the Psoas SUV_mean_ was similar to the intra-center variability of the Psoas SUV_mean_ (0.055 vs. 0.045, p=0.31). This prompted us to use the kidney/psoas mSUVRatio as an attempt of intra-individual “normalization”. Still, the multicenter design inherently introduced heterogeneity (e.g. different PET/CT devices, analog vs digital technologies, acquisition and reconstruction protocols), which may affect quantitative FDG uptake measurements. Following your advice of “harmonization” (see infra), we have calculated the Z-scores in our multicenter cohort. For each center, we have calculated the mean (m) and standard deviation (SD) of the 3 PET parameters: renal mSUV_mean_; psoas SUV_mean_; mSUVR. For each parameter in the center, we have calculated Z, using the equation (parameter – m) / SD. The Z-score is a standardized statistical measure, i.e. the mean and standard deviation of Z are 0 and 1. It quantifies the number of standard deviations by which an observation differs from the mean of a distribution.

In univariate analysis, the risk of non-normal histology (n=27, including Borderline and SCR) increased with the Z-score of the mSUVR: For each increase of 1 standard deviation (i.e. 0.86 for UZA-DIGITAL and UZB-ANALOGIC; 0.51 for CHU-ANALOGIC; and 0.72 for CHU-DIGITAL), the risk of abnormal histology is multiplied by 1.64 (p=0.014) (**Supplementary Table 2**). In multivariate models, the Z-score of mSUVR was significantly associated with the risk of non-normal histology (OR=1.542 [1.021–2.328, p=0.04), only after adjustment for the age of the donor (OR= 1.051 [1.008–1.095], p=0.019). The [18F]-FDG uptake reflects metabolic activity of inflammatory cells. Subclinical rejection, by definition, may involve limited or focal inflammation, potentially below the detection threshold of PET/CT imaging. Indeed, our multicenter cohort includes a broader spectrum of lesions, including the classical T-cell–mediated rejection and the recently Banff-recognized entity “microvascular inflammation (MVI) without DSA or C4d”. These lesions may involve different inflammatory patterns. As a whole, the monocentric studies (PMID: 31841263; PMID: 26302136; PMID: 34191101) may reflect a more homogeneous and selected population (distinguishing per-protocol *versus* per-cause biopsies, as an example), whereas the present multicentric cohort better reflects real-world heterogeneity, leading to reduced diagnostic performance of [18F]-FDG PET/CT imaging.

Explaining the discordant results between the initial monocentric study and the present multicenter investigation is challenging, despite the more than two‑fold increase in sample size. However, several observations can be made: In the pilot study mean mSUVR values were used, whereas the multicenter study appears to have relied on median values, introducing a potential inconsistency in the statistical treatment of the same parameter.

We thank Reviewer #2 for pointing out this possible source of confusion. In both the pilot monocentric study (PMID: 31841263) and the present multicenter study, the mean mSUVR values were used. First, the SUVmean was manually measured in 4 1-ml volumes of interest in the renal cortex and averaged to obtain the mean SUVmean of [18F]-FDG accumulation in the graft. Next, the SUVmean was measured in 1 20-ml volume of interest of the psoas muscle, considered as the reference tissue of the corresponding patient. Finally, the kidney/psoas ratio was individually calculated by dividing the mean of renal SUVmean by the psoas SUVmean: mSUVR. As their distribution is not Normal, the median values of these parameters are reported in Tables and the logarithm is used in the models.

Second, mSUVR measurements may vary across different PET scanner models, and cross‑calibration procedures should be applied not only to center‑specific devices but uniformly across all scanners involved in the study to ensure harmonization.

We thank Reviewer #2 for emphasizing the impact of harmonization across the different PET scanner models. Image acquisition and reconstruction were pragmatically done in real-life settings. Cross-calibration between the different PET/CT devices was not feasible for the specific purpose of the present study. Still, all PET/CT devices are routinely calibrated and EARL-certified. The quantification of renal [18F]-FDG uptake was performed manually following a standardized protocol. We have previously showed that the intra-class correlation coefficient ICC for inter-observer reproducibility for each VOI was 0.87 [0.81-0.91], 0.87 [0.81-0.91], 0.85 [0.78-0.89] and 0.83 [0.76-0.88] for the upper to the lower renal poles, respectively (PMID: 32165653). Furthermore, renal [18F]-FDG uptake was individually normalized to [18F]-FDG uptake by the psoas muscle of the patient. Finally, following your suggestions, we have calculated the z-scores in our multicenter cohort (see supra). Additional PET-derived metrics, such as metabolic tumor volume (MTV), would be pertinent but require multidimensional procedures of analysis, including automated segmentation of the renal graft standardized for the 4 PET/CT devices used in the present multicenter study. These highly relevant PET-derived metrics require further investigations, as detailed in the Discussion.

Third, the authors might consider evaluating additional PET‑derived metrics—such as metabolic tumor volume or other volumetric/metabolic parameters—which could potentially offer improved diagnostic performance.

We fully agree with Reviewer #2: additional PET-derived metrics, such as metabolic tumor volume (MTV), would be highly pertinent and could offer improved diagnostic performance. Still, such a sophisticated approach requires multidimensional procedures of analysis, including automated segmentation of the renal graft standardized for the 4 PET/CT devices used in the present multicenter study. These highly relevant PET-derived metrics require future investigations, as detailed in the Discussion. Still, on the basis of this thoughtful and motivating suggestion, we propose to nuance the title of the present manuscript focusing on the mSUVR, as follows: “A multicenter prospective validation cohort does not confirm the use of kidney/psoas [18F]FDG uptake in the diagnosis of kidney allograft subclinical rejection”.

Finally, a revised mSUVR threshold could be recalculated and validated in new patient cohorts to determine whether alternative cut‑offs may yield better discriminatory accuracy.

We thank Reviewer #2 for prompting us to revise the mSUVR threshold based on the present multicenter cohort including 185 patients. This data has been added to the Discussion.

|  |  |  |  |  |  |
| --- | --- | --- | --- | --- | --- |
| Variable | Cut-off Youden | SE (IC95%) | SP (IC95%) | VPP (IC95%) | VPN (IC95%) |
| Kidney/Muscle Mean SUVMean | **≥ 2.12** | 88.9 (51.8-99.7) | 33.5 (26.6-41.0) | 6.4 (2.8-12.2) | 98.3 (91.1-99.9) |

6. PLOS authors have the option to publish the peer review history of their article ([what does this mean?](https://track.editorialmanager.com/CL0/https:%2F%2Fjournals.plos.org%2Fplosone%2Fs%2Feditorial-and-peer-review-process%23loc-peer-review-history/1/010f019d8a28ac09-5d30fefd-7a74-4f3b-a178-85ea92d71cc3-000000/-WIBNT8QIPhSbHQjrcSpDUNMFhd69XfR4fe-E5s7Zfw=258)). If published, this will include your full peer review and any attached files.


If you choose “no”, your identity will remain anonymous but your review may still be made public.


**Do you want your identity to be public for this peer review?** For information about this choice, including consent withdrawal, please see our [Privacy Policy](https://track.editorialmanager.com/CL0/https:%2F%2Fwww.plos.org%2Fprivacy-policy/1/010f019d8a28ac09-5d30fefd-7a74-4f3b-a178-85ea92d71cc3-000000/z1ccD0hnUs082HqVpXv5oeMhMsx7fjpVQdA8KGh2LZw=258).

Reviewer #1: No

Reviewer #2: No

[NOTE: If reviewer comments were submitted as an attachment file, they will be attached to this email and accessible via the submission site. Please log into your account, locate the manuscript record, and check for the action link "View Attachments". If this link does not appear, there are no attachment files.]

To ensure your figures meet our technical requirements, please review our figure guidelines: [https://journals](https://journals/).plos.org/plosone/s/figures

You may also use PLOS’s free figure tool, NAAS, to help you prepare publication quality figures: [https://journals](https://journals/).plos.org/plosone/s/figures#loc-tools-for-figure-preparation.

NAAS will assess whether your figures meet our technical requirements by comparing each figure against our figure specifications.
