# Supplementary material for: A multicenter prospective validation cohort does not support the use of kidney/psoas [18F]FDG uptake in the diagnosis of kidney allograft subclinical rejection
Source: PLoS One. 2026 Sep 2;21(9):e0345426. doi: 10.1371/journal.pone.0345426 (PMC13537687; doi:10.1371/journal.pone.0345426)
Supplement: Peer review history

# Peer review: Decision Letter 0

**DOI:** 10.1371/journal.pone.0345426.r001

**Authors:** Carmelo Caldarella

14 Apr 2026

Dear Dr. Jouret,

Thank you for submitting your manuscript to PLOS ONE. After careful consideration, we feel that it has merit but does not fully meet PLOS ONE’s publication criteria as it currently stands. Therefore, we invite you to submit a revised version of the manuscript that addresses the points raised during the review process.

==============================

**ACADEMIC EDITOR:** **Main concerns: Minor concerns:**  please analyze additional PET-derived metrics as MTV or TLG; please specify better whether harmonization procedures were applied to overcome the drawbacks from obtaining functional measures from different scanners.

==============================

Please submit your revised manuscript by May 29 2026 11:59PM. If you will need more time than this to complete your revisions, please reply to this message or contact the journal office at plosone@plos.org. When you're ready to submit your revision, log on to https://www.editorialmanager.com/pone/ and select the 'Submissions Needing Revision' folder to locate your manuscript file.

- A letter that responds to each point raised by the academic editor and reviewer(s). You should upload this letter as a separate file labeled 'Response to Reviewers'.
- A marked-up copy of your manuscript that highlights changes made to the original version. You should upload this as a separate file labeled 'Revised Manuscript with Track Changes'.
- An unmarked version of your revised paper without tracked changes. You should upload this as a separate file labeled 'Manuscript'.

If you would like to make changes to your financial disclosure, please include your updated statement in your cover letter. Guidelines for resubmitting your figure files are available below the reviewer comments at the end of this letter.

If applicable, we recommend that you deposit your laboratory protocols in protocols.io to enhance the reproducibility of your results. Protocols.io assigns your protocol its own identifier (DOI) so that it can be cited independently in the future. For instructions see: https://journals.plos.org/plosone/s/submission-guidelines#loc-laboratory-protocols. Additionally, PLOS ONE offers an option for publishing peer-reviewed Lab Protocol articles, which describe protocols hosted on protocols.io. Read more information on sharing protocols at https://plos.org/protocols?utm_medium=editorial-email&utm_source=authorletters&utm_campaign=protocols.

As the corresponding author, your ORCID iD is verified in the submission system and will appear in the published article. PLOS supports the use of ORCID, and we encourage all coauthors to register for an ORCID iD and use it as well. Please encourage your coauthors to verify their ORCID iD within the submission system before final acceptance, as unverified ORCID iDs will not appear in the published article. *Only*  the individual author can complete the verification step; PLOS staff *cannot*  verify ORCID iDs on behalf of authors.

We look forward to receiving your revised manuscript.

Kind regards,

Carmelo Caldarella, Ph.D., M.D.

Academic Editor

PLOS One

Journal Requirements:

When submitting your revision, we need you to address these additional requirements.

1. Please ensure that your manuscript meets PLOS ONE's style requirements, including those for file naming. The PLOS ONE style templates can be found at

https://journals.plos.org/plosone/s/file?id=wjVg/PLOSOne_formatting_sample_main_body.pdf and https://journals.plos.org/plosone/s/file?id=ba62/PLOSOne_formatting_sample_title_authors_affiliations.pdf

2. We note that you have indicated that there are restrictions to data sharing for this study. For studies involving human research participant data or other sensitive data, we encourage authors to share de-identified or anonymized data. However, when data cannot be publicly shared for ethical reasons, we allow authors to make their data sets available upon request. For information on unacceptable data access restrictions, please see http://journals.plos.org/plosone/s/data-availability#loc-unacceptable-data-access-restrictions.

Before we proceed with your manuscript, please address the following prompts:

a) If there are ethical or legal restrictions on sharing a de-identified data set, please explain them in detail (e.g., data contain potentially identifying or sensitive patient information, data are owned by a third-party organization, etc.) and who has imposed them (e.g., a Research Ethics Committee or Institutional Review Board, etc.). Please also provide contact information for a data access committee, ethics committee, or other institutional body to which data requests may be sent.

b) If there are no restrictions, please upload the minimal anonymized data set necessary to replicate your study findings to a stable, public repository and provide us with the relevant URLs, DOIs, or accession numbers. Please see http://www.bmj.com/content/340/bmj.c181.long for guidelines on how to de-identify and prepare clinical data for publication. For a list of recommended repositories, please see https://journals.plos.org/plosone/s/recommended-repositories. You also have the option of uploading the data as Supporting Information files, but we would recommend depositing data directly to a data repository if possible.

Please update your Data Availability statement in the submission form accordingly.

3. Thank you for stating the following in the Acknowledgments Section of your manuscript:

The authors cordially thank the patients-participants, the surgeons (A. De Roover, O. Detry, N. Gilbo, N. Meurisse, and M. Vandermeulen), the physicians (L. Vanovermeire and P. Xhignesse), and the members of the local transplant coordination center (J Mornard, F. Sinte and A Warmoes) for their commitment to kidney transplantation at the University of Liege Hospital in Liege, Belgium; the surgeons (T. Chapelle, G. Roeyen, B. Bracke, V. Hartman, B. Hendrikx, E. Liekens), the physicians (M. Dirix, H. de Fijter, V. de Meier), the study coordinator (P. Lievens) and transplant coordinators (G. Van Beeumen, P. Hollants, A. Van de Weyer, L. Van den Bergh, M. De Deyne) at Universitair Ziekenhuis Antwerpen in Antwerp, Belgium; the surgeons, the physicians, the study coordinator and the transplant coordinators at Universitair Ziekenhuis Brussel in Brussels, Belgium. AB is a Fellow of the Fonds National de la Recherche Scientifique. FJ and RH received support from the University of Liège (Fonds Spéciaux à la Recherche; Fondation Léon Fredericq), as well as from ULiège Academic Hospital (Fonds d’Investissement pour la Recherche Scientifique, FIRS). This study was supported by the Belgian Transplant Society.

We note that you have provided funding information that is not currently declared in your Funding Statement. However, funding information should not appear in the Acknowledgments section or other areas of your manuscript. We will only publish funding information present in the Funding Statement section of the online submission form.

Please remove any funding-related text from the manuscript and let us know how you would like to update your Funding Statement. Currently, your Funding Statement reads as follows:

FJ and RH received support from the University of Liège (Fonds Spéciaux à la Recherche; Fondation Léon Fredericq), as well as from ULiège Academic Hospital (Fonds d’Investissement pour la Recherche Scientifique, FIRS). This study was supported by the Belgian Transplant Society.

Please include your amended statements within your cover letter; we will change the online submission form on your behalf.

4. Please include captions for your Supporting Information files at the end of your manuscript, and update any in-text citations to match accordingly. Please see our Supporting Information guidelines for more information: http://journals.plos.org/plosone/s/supporting-information.

5. If the reviewer comments include a recommendation to cite specific previously published works, please review and evaluate these publications to determine whether they are relevant and should be cited. There is no requirement to cite these works unless the editor has indicated otherwise.

[Note: HTML markup is below. Please do not edit.]

Reviewers' comments:

Reviewer's Responses to Questions

**Comments to the Author**

1. Is the manuscript technically sound, and do the data support the conclusions?

Reviewer #1: Partly

Reviewer #2: Yes

**********

2. Has the statistical analysis been performed appropriately and rigorously?

Reviewer #1: No

Reviewer #2: Yes

**********

3. Have the authors made all data underlying the findings in their manuscript fully available?

The PLOS Data policy

Reviewer #1: No

Reviewer #2: Yes

**********

4. Is the manuscript presented in an intelligible fashion and written in standard English?

Reviewer #1: No

Reviewer #2: Yes

**********

Reviewer #1: The study addressed an important topic; however, several areas required revision to meet publication standards. Methodological transparency should be improved, particularly regarding participant selection, experimental procedures, and controls. Statistical reporting should be more comprehensive and aligned with standard guidelines. The discussion should better integrate existing literature and clearly acknowledge study limitations, including potential bias and generalizability concerns. Figures and tables should be refined with clearer legends and consistent formatting. Ethical considerations and approvals, if applicable, should be explicitly stated. Overall, substantial revision was required to enhance clarity, reproducibility, and scientific STANDARD

Reviewer #2: In this multicenter prospective investigation, the authors aimed to assess the diagnostic reliability of [18F]FDG PET/CT as a non‑invasive screening modality for the exclusion of subclinical acute kidney allograft rejection (SCR). This study resembles a previous article published in 2019, in which most of the current authors demonstrated the usefulness of FDG PET/CT for excluding SCR in a cohort of 92 patients. However, after applying the same PET‑derived parameter—the mean SUV ratio between the renal cortex and the psoas muscle (mSUVR)—the present study found no significant correlation with the t‑i histological score, nor was mSUVR associated with an increased risk of non‑normal histology in multivariate analysis. The authors therefore conclude that this metric, tested in a larger cohort of 185 patients, is not effective in distinguishing normal, borderline, and SCR cases, and does not provide sufficient diagnostic yield to rule out SCR in clinically stable recipients at 3 months after kidney transplantation.

Explaining the discordant results between the initial monocentric study and the present multicenter investigation is challenging, despite the more than two‑fold increase in sample size. However, several observations can be made.

In the pilot study mean mSUVR values were used, whereas the multicenter study appears to have relied on median values, introducing a potential inconsistency in the statistical treatment of the same parameter.

Second, mSUVR measurements may vary across different PET scanner models, and cross‑calibration procedures should be applied not only to center‑specific devices but uniformly across all scanners involved in the study to ensure harmonization.

Third, the authors might consider evaluating additional PET‑derived metrics—such as metabolic tumor volume or other volumetric/metabolic parameters—which could potentially offer improved diagnostic performance.

Finally, a revised mSUVR threshold could be recalculated and validated in new patient cohorts to determine whether alternative cut‑offs may yield better discriminatory accuracy.

**********

what does this mean?). If published, this will include your full peer review and any attached files.

If you choose “no”, your identity will remain anonymous but your review may still be made public.

**Do you want your identity to be public for this peer review?**  For information about this choice, including consent withdrawal, please see our Privacy Policy

Reviewer #1: No

Reviewer #2: No

**********

[NOTE: If reviewer comments were submitted as an attachment file, they will be attached to this email and accessible via the submission site. Please log into your account, locate the manuscript record, and check for the action link "View Attachments". If this link does not appear, there are no attachment files.]

To ensure your figures meet our technical requirements, please review our figure guidelines: https://journals.plos.org/plosone/s/figures

You may also use PLOS’s free figure tool, NAAS, to help you prepare publication quality figures: https://journals.plos.org/plosone/s/figures#loc-tools-for-figure-preparation.

NAAS will assess whether your figures meet our technical requirements by comparing each figure against our figure specifications.

# Author comment: Author response to Decision Letter 1

**DOI:** 10.1371/journal.pone.0345426.r002

27 May 2026

PONE-D-26-10474

A multicenter prospective validation cohort does not confirm the diagnostic yield of [18F]FDG PET/CT imaging in kidney allograft subclinical rejection

PLOS One

ACADEMIC EDITOR:

Main concerns: please specify better what makes your paper different from similar research available currently in the literature, and why your results are somehow in contrast with published evidence

We would like to thank the Academic Editor for emphasizing that our prospective multicenter cohort study has merit and inviting us to submit a revised version of the manuscript including all suggestions and comments of Reviewers #1 and #2. Especially, we have better discussed what makes our paper different from similar research available currently in the literature, and why our results are somehow in contrast with published evidence. The pilot study (PMID: 31841263) included only 6 cases of subclinical rejection, which may have led to an overestimation of diagnostic performance and instability of the proposed 2.4 mSUVR threshold. Furthermore, this pilot study was monocentric. Focusing on the 158 cases with biopsy-proven normal histology of the present multicenter cohort, the inter-center variability of the Kidney SUVmean was greater than intra-center variability of the Kidney SUVmean (0.91 vs. 0.14, p=0.0003). By contrast, the inter-center variability of the Psoas SUVmean was similar to the intra-center variability of the Psoas SUVmean (0.055 vs. 0.045, p=0.31). This prompted us to use the kidney/psoas mSUVRatio as an attempt of intra-individual “normalization”. Still, the multicenter design inherently introduced heterogeneity (e.g. different PET/CT devices, analog vs digital technologies, acquisition and reconstruction protocols), which may affect quantitative FDG uptake measurements. Following the advice of “harmonization” of Reviewer #2, we have calculated the Z-scores in our multicenter cohort. For each center, we have calculated the mean (m) and standard deviation (SD) of the 3 PET parameters: renal mSUVmean; psoas SUVmean; mSUVR. For each parameter in the center, we have calculated Z, using the equation (parameter – m) / SD. The Z-score is a standardized statistical measure, i.e. the mean and standard deviation of Z are 0 and 1. It quantifies the number of standard deviations by which an observation differs from the mean of a distribution.

In univariate analysis, the risk of non-normal histology (n=27, including Borderline and SCR) increased with the Z-score of the mSUVR: For each increase of 1 standard deviation (i.e. 0.86 for UZA-DIGITAL and UZB-ANALOGIC; 0.51 for CHU-ANALOGIC; and 0.72 for CHU-DIGITAL), the risk of abnormal histology is multiplied by 1.64 (p=0.014) (Supplementary Table 2). In multivariate models, the Z-score of mSUVR was significantly associated with the risk of non-normal histology (OR=1.542 [1.021–2.328, p=0.04). The [18F]-FDG uptake reflects metabolic activity of inflammatory cells. Subclinical rejection, by definition, may involve limited or focal inflammation, potentially below the detection threshold of PET/CT imaging. Indeed, our multicenter cohort includes a broader spectrum of lesions, including the classical T-cell–mediated rejection and the recently Banff-recognized entity “microvascular inflammation (MVI) without DSA or C4d”. These lesions may involve different inflammatory patterns. As a whole, the monocentric studies (PMID: 31841263; PMID: 26302136; PMID: 34191101) may reflect a more homogeneous and selected population (distinguishing per-protocol versus per-cause biopsies, as an example), whereas the present multicentric cohort better reflects real-world heterogeneity, leading to reduced diagnostic performance of [18F]-FDG PET/CT imaging.

Minor concerns: please analyze additional PET-derived metrics as MTV or TLG; please specify better whether harmonization procedures were applied to overcome the drawbacks from obtaining functional measures from different scanners.

We would like to thank the Academic Editor and Reviewer #2 for helping us to strengthen the harmonization process of our multicenter data. Image acquisition and reconstruction were pragmatically done in real-life settings. Cross-calibration between the different PET/CT devices was not feasible for the specific purpose of the present study. Still, all PET/CT devices are routinely calibrated and EARL-certified. The quantification of renal [18F]-FDG uptake was performed manually following a standardized protocol. We have previously showed that the intra-class correlation coefficient ICC for inter-observer reproducibility for each VOI was 0.87 [0.81-0.91], 0.87 [0.81-0.91], 0.85 [0.78-0.89] and 0.83 [0.76-0.88] for the upper to the lower renal poles, respectively (PMID: 32165653). Furthermore, renal [18F]-FDG uptake was individually normalized to [18F]-FDG uptake by the psoas muscle of the patient. Finally, following the suggestions of Reviewer #2, we have calculated the z-scores in our multicenter cohort (see supra). Additional PET-derived metrics, such as metabolic tumor volume (MTV), would be pertinent but require multidimensional procedures of analysis, including automated segmentation of the renal graft standardized for the 4 PET/CT devices used in the present multicenter study. These highly relevant PET-derived metrics require further investigations, as detailed in the Discussion.

On the basis of the comments and suggestions of the Academic Editor and the Reviewers, we propose to scientifically nuance the title of the manuscript focusing on the mSUVR, as follows: “A multicenter prospective validation cohort does not support the use of kidney/psoas [18F]FDG uptake in the diagnosis of kidney allograft subclinical rejection”.

==============================

Reviewer #1: The study addressed an important topic; however, several areas required revision to meet publication standards.

We would like to thank Reviewer #1 for emphasizing that our study addresses an important topic.

Methodological transparency should be improved, particularly regarding participant selection, experimental procedures, and controls.

The methodology, particularly regarding participant selection, experimental procedures, and controls, has been further detailed in the “Patients and Methods” section of the revised version of the manuscript.

Statistical reporting should be more comprehensive and aligned with standard guidelines.

The statistical methods have been more detailed in the Methods section of the revised manuscript.

The discussion should better integrate existing literature and clearly acknowledge study limitations, including potential bias and generalizability concerns.

We have better discussed what makes our paper different from similar research available currently in the literature, and why our results are somehow in contrast with published evidence. The pilot study (PMID: 31841263) included only 6 cases of subclinical rejection, which may have led to an overestimation of diagnostic performance and instability of the proposed 2.4 mSUVR threshold. Furthermore, this pilot study was monocentric. Focusing on the 158 cases with biopsy-proven normal histology of the present multicenter cohort, the inter-center variability of the Kidney SUVmean was greater than intra-center variability of the Kidney SUVmean (0.91 vs. 0.14, p=0.0003). By contrast, the inter-center variability of the Psoas SUVmean was similar to the intra-center variability of the Psoas SUVmean (0.055 vs. 0.045, p=0.31). This prompted us to use the kidney/psoas mSUVRatio as an attempt of intra-individual “normalization”. Still, the multicenter design inherently introduced heterogeneity (e.g. different PET/CT devices, analog vs digital technologies, acquisition and reconstruction protocols), which may affect quantitative FDG uptake measurements. Following the advice of “harmonization” of Reviewer #2, we have calculated the Z-scores in our multicenter cohort. For each center, we have calculated the mean (m) and standard deviation (SD) of the 3 PET parameters: renal mSUVmean; psoas SUVmean; mSUVR. For each parameter in the center, we have calculated Z, using the equation (parameter – m) / SD. The Z-score is a standardized statistical measure, i.e. the mean and standard deviation of Z are 0 and 1. It quantifies the number of standard deviations by which an observation differs from the mean of a distribution.

In univariate analysis, the risk of non-normal histology (n=27, including Borderline and SCR) increased with the Z-score of the mSUVR: For each increase of 1 standard deviation (i.e. 0.86 for UZA-DIGITAL and UZB-ANALOGIC; 0.51 for CHU-ANALOGIC; and 0.72 for CHU-DIGITAL), the risk of abnormal histology is multiplied by 1.64 (p=0.014) (Supplementary Table 2). In multivariate models, the Z-score of mSUVR was significantly associated with the risk of non-normal histology (OR=1.542 [1.021–2.328, p=0.04), only after adjustment for the age of the donor (OR= 1.051 [1.008–1.095], p=0.019). The [18F]-FDG uptake reflects metabolic activity of inflammatory cells. Subclinical rejection, by definition, may involve limited or focal inflammation, potentially below the detection threshold of PET/CT imaging. Indeed, our multicenter cohort includes a broader spectrum of lesions, including the classical T-cell–mediated rejection and the recently Banff-recognized entity “microvascular inflammation (MVI) without DSA or C4d”. These lesions may involve different inflammatory patterns. As a whole, the monocentric studies (PMID: 31841263; PMID: 26302136; PMID: 34191101) may reflect a more homogeneous and selected population (distinguishing per-protocol versus per-cause biopsies, as an example), whereas the present multicentric cohort better reflects real-world heterogeneity, leading to reduced diagnostic performance of [18F]-FDG PET/CT imaging.

Figures and tables should be refined with clearer legends and consistent formatting.

We thank Reviewer #1 for referring to the standardized formatting of Figures and Legends according to PLOS One instructions.

Ethical considerations and approvals, if applicable, should be explicitly stated.

This study has been approved by the Institutional Review Board (IRB) of CHULiège under the number B707202042977, and endorsed by the IRB of UZA and UZB. All these documents have been scanned and downloaded on the PLOS One submission website. Kidney transplantations were performed in accordance with the Declaration of Istanbul.

Overall, substantial revision was required to enhance clarity, reproducibility, and scientific STANDARD

We would like to thank Reviewer #1 for helping us to improve our manuscript according to the scientific standards and instructions of PLOS One.

Reviewer #2: In this multicenter prospective investigation, the authors aimed to assess the diagnostic reliability of [18F]FDG PET/CT as a non invasive screening modality for the exclusion of subclinical acute kidney allograft rejection (SCR). This study resembles a previous article published in 2019, in which most of the current authors demonstrated the usefulness of FDG PET/CT for excluding SCR in a cohort of 92 patients. However, after applying the same PET derived parameter—the mean SUV ratio between the renal cortex and the psoas muscle (mSUVR)—the present study found no significant correlation with the t i histological score, nor was mSUVR associated with an increased risk of non normal histology in multivariate analysis. The authors therefore conclude that this metric, tested in a larger cohort of 185 patients, is not effective in distinguishing normal, borderline, and SCR cases, and does not provide sufficient diagnostic yield to rule out SCR in clinically stable recipients at 3 months after kidney transplantation.

We would like to thank Reviewer #2 for his/her constructive suggestions and comments about our prospective multicenter cohort study.

We have better discussed what makes our paper different from similar research available currently in the literature, and why our results are somehow in contrast with published evidence. The pilot study (PMID: 31841263) included only 6 cases of subclinical rejection, which may have led to an overestimation of diagnostic performance and instability of the proposed 2.4 mSUVR threshold. Furthermore, this pilot study was monocentric. Focusing on the 158 cases with biopsy-proven normal histology of the present multicenter cohort, the inter-center variability of the Kidney SUVmean was greater than intra-center variability of the Kidney SUVmean (0.91 vs. 0.14, p=0.0003). By contrast, the inter-center variability of the Psoas SUVmean was similar to the intra-center variability of the Psoas SUVmean (0.055 vs. 0.045, p=0.31). This prompted us to use the kidney/psoas mSUVRatio as an attempt of intra-individual “normalization”. Still, the multicenter design inherently introduced heterogeneity (e.g. different PET/CT devices, analog vs digital technologies, acquisition and reconstruction protocols), which may affect quantitative FDG uptake measurements. Following your advice of “harmonization” (see infra), we have calculated the Z-scores in our multicenter cohort. For each center, we have calculated the mean (m) and standard deviation (SD) of the 3 PET parameters: renal mSUVmean; psoas SUVmean; mSUVR. For each parameter in the center, we have calculated Z, using the equation (parameter – m) / SD. The Z-score is a standardized statistical measure, i.e. the mean and standard deviation of Z are 0 and 1. It quantifies the number of standard deviations by which an observation differs from the mean of a distribution.

In univariate analysis, the risk of non-normal histology (n=27, including Borderline and SCR) increased with the Z-score of the mSUVR: For each increase of 1 standard deviation (i.e. 0.86 for UZA-DIGITAL and UZB-ANALOGIC; 0.51 for CHU-ANALOGIC; and 0.72 for CHU-DIGITAL), the risk of abnormal histology is multiplied by 1.64 (p=0.014) (Supplementary Table 2). In multivariate models, the Z-score of mSUVR was significantly associated with the risk of non-normal histology (OR=1.542 [1.021–2.328, p=0.04), only after adjustment for the age of the donor (OR= 1.051 [1.008–1.095], p=0.019). The [18F]-FDG uptake reflects metabolic activity of inflammatory cells. Subclinical rejection, by definition, may involve limited or focal inflammation, potentially below the detection threshold of PET/CT imaging. Indeed, our multicenter cohort includes a broader spectrum of lesions, including the classical T-cell–mediated rejection and the recently Banff-recognized entity “microvascular inflammation (MVI) without DSA or C4d”. These lesions may involve different inflammatory patterns. As a whole, the monocentric studies (PMID: 31841263; PMID: 26302136; PMID: 34191101) may reflect a more homogeneous and selected population (distinguishing per-protocol versus per-cause biopsies, as an example), whereas the present multicentric cohort better reflects real-world heterogeneity, leading to reduced diagnostic performance of [18F]-FDG PET/CT imaging.

Explaining the discordant results between the initial monocentric study and the present multicenter investigation is challenging, despite the more than two fold increase in sample size. However, several observations can be made: In the pilot study mean mSUVR values were used, whereas the multicenter study appears to have relied on median values, introducing a potential inconsistency in the statistical treatment of the same parameter.

We thank Reviewer #2 for pointing out this possible source of confusion. In both the pilot monocentric study (PMID: 31841263) and the present multicenter study, the mean mSUVR values were used. First, the SUVmean was manually measured in 4 1-ml volumes of interest in the renal cortex and averaged to obtain the mean SUVmean of [18F]-FDG accumulation in the graft. Next, the SUVmean was measured in 1 20-ml volume of interest of the psoas muscle, considered as the reference tissue of the corresponding patient. Finally, the kidney/psoas ratio was individually calculated by dividing the mean of renal SUVmean by the psoas SUVmean: mSUVR. As their distribution is not Normal, the median values of these parameters are reported in Tables and the logarithm is used in the models.

Second, mSUVR measurements may vary acro

Attachment Submitted filename: PETCT_SCR_PLOSOne_ResponseToReviewers_R1.docx

# Peer review: Decision Letter 1

**DOI:** 10.1371/journal.pone.0345426.r003

**Authors:** Carmelo Caldarella

16 Jul 2026

Dear Dr. Jouret,

Thank you for submitting your manuscript to PLOS ONE. After careful consideration, we feel that it has merit but does not fully meet PLOS ONE’s publication criteria as it currently stands. Therefore, we invite you to submit a revised version of the manuscript that addresses the points raised during the review process.

==============================

**ACADEMIC EDITOR:** **Minor issue:**

Please submit your revised manuscript by Aug 30 2026 11:59PM. If you will need more time than this to complete your revisions, please reply to this message or contact the journal office at plosone@plos.org. When you're ready to submit your revision, log on to https://www.editorialmanager.com/pone/ and select the 'Submissions Needing Revision' folder to locate your manuscript file.

- A letter that responds to each point raised by the academic editor and reviewer(s). You should upload this letter as a separate file labeled 'Response to Reviewers'.
- A marked-up copy of your manuscript that highlights changes made to the original version. You should upload this as a separate file labeled 'Revised Manuscript with Track Changes'.
- An unmarked version of your revised paper without tracked changes. You should upload this as a separate file labeled 'Manuscript'.

If you would like to make changes to your financial disclosure, please include your updated statement in your cover letter. Guidelines for resubmitting your figure files are available below the reviewer comments at the end of this letter.

If applicable, we recommend that you deposit your laboratory protocols in protocols.io to enhance the reproducibility of your results. Protocols.io assigns your protocol its own identifier (DOI) so that it can be cited independently in the future. For instructions see: https://journals.plos.org/plosone/s/submission-guidelines#loc-laboratory-protocols. Additionally, PLOS ONE offers an option for publishing peer-reviewed Lab Protocol articles, which describe protocols hosted on protocols.io. Read more information on sharing protocols at https://plos.org/protocols?utm_medium=editorial-email&utm_source=authorletters&utm_campaign=protocols.

As the corresponding author, your ORCID iD is verified in the submission system and will appear in the published article. PLOS supports the use of ORCID, and we encourage all coauthors to register for an ORCID iD and use it as well. Please encourage your coauthors to verify their ORCID iD within the submission system before final acceptance, as unverified ORCID iDs will not appear in the published article. *Only*  the individual author can complete the verification step; PLOS staff *cannot*  verify ORCID iDs on behalf of authors.

We look forward to receiving your revised manuscript.

Kind regards,

Carmelo Caldarella, Ph.D., M.D.

Academic Editor

PLOS One

Journal Requirements:

If the reviewer comments include a recommendation to cite specific previously published works, please review and evaluate these publications to determine whether they are relevant and should be cited. There is no requirement to cite these works unless the editor has indicated otherwise.

Please review your reference list to ensure that it is complete and correct. If you have cited papers that have been retracted, please include the rationale for doing so in the manuscript text, or remove these references and replace them with relevant current references. Any changes to the reference list should be mentioned in the rebuttal letter that accompanies your revised manuscript. If you need to cite a retracted article, indicate the article’s retracted status in the References list and also include a citation and full reference for the retraction notice.

[Note: HTML markup is below. Please do not edit.]

Reviewers' comments:

Reviewer's Responses to Questions

**Comments to the Author**

Reviewer #1: All comments have been addressed

Reviewer #2: All comments have been addressed

**********

2. Is the manuscript technically sound, and do the data support the conclusions?

Reviewer #1: Partly

Reviewer #2: Yes

**********

3. Has the statistical analysis been performed appropriately and rigorously?

Reviewer #1: Yes

Reviewer #2: Yes

**********

4. Have the authors made all data underlying the findings in their manuscript fully available?

The PLOS Data policy

Reviewer #1: Yes

Reviewer #2: Yes

**********

5. Is the manuscript presented in an intelligible fashion and written in standard English?

Reviewer #1: Yes

Reviewer #2: Yes

**********

Reviewer #1: Major Comments

The number of biopsy-proven subclinical rejection cases was very small (n=9), which substantially limited statistical power. This limitation should be emphasized more clearly in the discussion.

The authors should provide additional diagnostic performance metrics (sensitivity, specificity, positive predictive value, negative predictive value, and ROC analysis) for the proposed mSUVR threshold to facilitate clinical interpretation.

PET/CT acquisitions were performed across different centers and scanners. Although stratified analyses were performed, the potential impact of inter-scanner variability and the absence of central image review should be discussed in greater detail.

Minor Comments

Several typographical and grammatical errors should be corrected throughout the manuscript.

The rationale for combining borderline lesions and rejection lesions in some analyses should be explained more clearly.

Figure legends could be expanded to improve clarity and facilitate interpretation without referring extensively to the main text.

The discussion may benefit from a brief comparison with emerging non-invasive biomarkers such as donor-derived cell-free DNA and molecular diagnostics.

Reviewer #2: (No Response)

**********

what does this mean?). If published, this will include your full peer review and any attached files.

If you choose “no”, your identity will remain anonymous but your review may still be made public.

**Do you want your identity to be public for this peer review?**  For information about this choice, including consent withdrawal, please see our Privacy Policy

Reviewer #1: No

Reviewer #2: **Yes:** Massimo CASTELLANI

**********

[NOTE: If reviewer comments were submitted as an attachment file, they will be attached to this email and accessible via the submission site. Please log into your account, locate the manuscript record, and check for the action link "View Attachments". If this link does not appear, there are no attachment files.]

To ensure your figures meet our technical requirements, please review our figure guidelines: https://journals.plos.org/plosone/s/figures

You may also use PLOS’s free figure tool, NAAS, to help you prepare publication quality figures: https://journals.plos.org/plosone/s/figures#loc-tools-for-figure-preparation.

NAAS will assess whether your figures meet our technical requirements by comparing each figure against our figure specifications.

# Author comment: Author response to Decision Letter 2

**DOI:** 10.1371/journal.pone.0345426.r004

24 Jul 2026

PONE-D-26-10474R1

A multicenter prospective validation cohort does not support the use of kidney/psoas [18F]FDG uptake in the diagnosis of kidney allograft subclinical rejection

PLOS One

Dear Dr. Jouret,

Thank you for submitting your manuscript to PLOS ONE. After careful consideration, we feel that it has merit but does not fully meet PLOS ONE’s publication criteria as it currently stands. Therefore, we invite you to submit a revised version of the manuscript that addresses the points raised during the review process.

==============================

ACADEMIC EDITOR:

Minor issue: address in the Discussion how the small number of biopsy-proven sub-clinical rejection cases has influenced statistical power.

We thank the Academic Editor and the Reviewer #1 for raising this important point. Indeed, the biopsy-proven SCR group remained small (n=9, including 6 TCMR and 3 MVI), despite our cohort of 185 patients exceeding the pre-specified sample size (n=156) calculated to detect a 7% SCR incidence with 80% power. This limited event count restricts the precision of estimates specific to the SCR subgroup, with wide confidence intervals around mSUVR values and diagnostic performance metrics. To partly address this, we combined SCR with borderline cases (n=27, i.e. 14.6%) for the primary multivariate analysis of "non-normal histology," which improved statistical power and allowed significant associations to emerge (e.g., Z-score of mSUVR, OR=1.542, p=0.04). Still, we have to admit that 5/6 TCMR showed mSUVR below the 2.4 threshold established by our previous study. We now explicitly acknowledge in the Discussion that the low SCR incidence, while consistent with contemporary cohorts, limits definitive conclusions regarding mSUVR's diagnostic yield specifically for SCR, and larger multicenter cohorts are needed for adequately powered SCR-specific analyses.

==============================

Please submit your revised manuscript by Aug 30 2026 11:59PM. If you will need more time than this to complete your revisions, please reply to this message or contact the journal office at plosone@plos.org. When you're ready to submit your revision, log on to https://www.editorialmanager.com/pone/ and select the 'Submissions Needing Revision' folder to locate your manuscript file.

Please include the following items when submitting your revised manuscript:

• A letter that responds to each point raised by the academic editor and reviewer(s). You should upload this letter as a separate file labeled 'Response to Reviewers'.

• A marked-up copy of your manuscript that highlights changes made to the original version. You should upload this as a separate file labeled 'Revised Manuscript with Track Changes'.

• An unmarked version of your revised paper without tracked changes. You should upload this as a separate file labeled 'Manuscript'.

If you would like to make changes to your financial disclosure, please include your updated statement in your cover letter. Guidelines for resubmitting your figure files are available below the reviewer comments at the end of this letter.

If applicable, we recommend that you deposit your laboratory protocols in protocols.io to enhance the reproducibility of your results. Protocols.io assigns your protocol its own identifier (DOI) so that it can be cited independently in the future. For instructions see: https://journals.plos.org/plosone/s/submission-guidelines#loc-laboratory-protocols. Additionally, PLOS ONE offers an option for publishing peer-reviewed Lab Protocol articles, which describe protocols hosted on protocols.io. Read more information on sharing protocols at https://plos.org/protocols?utm_medium=editorial-email&utm_source=authorletters&utm_campaign=protocols.

As the corresponding author, your ORCID iD is verified in the submission system and will appear in the published article. PLOS supports the use of ORCID, and we encourage all coauthors to register for an ORCID iD and use it as well. Please encourage your coauthors to verify their ORCID iD within the submission system before final acceptance, as unverified ORCID iDs will not appear in the published article. Only the individual author can complete the verification step; PLOS staff cannot verify ORCID iDs on behalf of authors.

We look forward to receiving your revised manuscript.

Kind regards,

Carmelo Caldarella, Ph.D., M.D.

Academic Editor

PLOS One

Journal Requirements:

If the reviewer comments include a recommendation to cite specific previously published works, please review and evaluate these publications to determine whether they are relevant and should be cited. There is no requirement to cite these works unless the editor has indicated otherwise.

Please review your reference list to ensure that it is complete and correct. If you have cited papers that have been retracted, please include the rationale for doing so in the manuscript text, or remove these references and replace them with relevant current references. Any changes to the reference list should be mentioned in the rebuttal letter that accompanies your revised manuscript. If you need to cite a retracted article, indicate the article’s retracted status in the References list and also include a citation and full reference for the retraction notice.

Reviewers' comments:

Reviewer's Responses to Questions

Comments to the Author

1. If the authors have adequately addressed your comments raised in a previous round of review and you feel that this manuscript is now acceptable for publication, you may indicate that here to bypass the “Comments to the Author” section, enter your conflict of interest statement in the “Confidential to Editor” section, and submit your "Accept" recommendation.

Reviewer #1: All comments have been addressed

Reviewer #2: All comments have been addressed

________________________________________

2. Is the manuscript technically sound, and do the data support the conclusions?

The manuscript must describe a technically sound piece of scientific research with data that supports the conclusions. Experiments must have been conducted rigorously, with appropriate controls, replication, and sample sizes. The conclusions must be drawn appropriately based on the data presented.

Reviewer #1: Partly

Reviewer #2: Yes

________________________________________

3. Has the statistical analysis been performed appropriately and rigorously?

Reviewer #1: Yes

Reviewer #2: Yes

________________________________________

4. Have the authors made all data underlying the findings in their manuscript fully available?

The PLOS Data policy requires authors to make all data underlying the findings described in their manuscript fully available without restriction, with rare exception (please refer to the Data Availability Statement in the manuscript PDF file). The data should be provided as part of the manuscript or its supporting information, or deposited to a public repository. For example, in addition to summary statistics, the data points behind means, medians and variance measures should be available. If there are restrictions on publicly sharing data—e.g. participant privacy or use of data from a third party—those must be specified.

Reviewer #1: Yes

Reviewer #2: Yes

________________________________________

5. Is the manuscript presented in an intelligible fashion and written in standard English?

PLOS ONE does not copyedit accepted manuscripts, so the language in submitted articles must be clear, correct, and unambiguous. Any typographical or grammatical errors should be corrected at revision, so please note any specific errors here.

Reviewer #1: Yes

Reviewer #2: Yes

________________________________________

6. Review Comments to the Author

Please use the space provided to explain your answers to the questions above. You may also include additional comments for the author, including concerns about dual publication, research ethics, or publication ethics. (Please upload your review as an attachment if it exceeds 20,000 characters)

Reviewer #1: Major Comments

The number of biopsy-proven subclinical rejection cases was very small (n=9), which substantially limited statistical power. This limitation should be emphasized more clearly in the discussion.

We thank the Academic Editor and the Reviewer #1 for raising this important point. Indeed, the biopsy-proven SCR group remained small (n=9, including 6 TCMR and 3 MVI), despite our cohort of 185 patients exceeding the pre-specified sample size (n=156) calculated to detect a 7% SCR incidence with 80% power. This limited event count restricts the precision of estimates specific to the SCR subgroup, with wide confidence intervals around mSUVR values and diagnostic performance metrics. To partly address this, we combined SCR with borderline cases (n=27, i.e. 14.6%) for the primary multivariate analysis of "non-normal histology," which improved statistical power and allowed significant associations to emerge (e.g., Z-score of mSUVR, OR=1.542, p=0.04). Still, we have to admit that 5/6 TCMR showed mSUVR below the 2.4 threshold established by our previous study. We now explicitly acknowledge in the Discussion that the low SCR incidence, while consistent with contemporary cohorts, limits definitive conclusions regarding mSUVR's diagnostic yield specifically for SCR, and larger multicenter cohorts are needed for adequately powered SCR-specific analyses.

The authors should provide additional diagnostic performance metrics (sensitivity, specificity, positive predictive value, negative predictive value, and ROC analysis) for the proposed mSUVR threshold to facilitate clinical interpretation.

The diagnostic performance metrics for the proposed mSUVR threshold (i.e. 2.12) were already mentioned in the Discussion: Lines 15-17, page 10.

PET/CT acquisitions were performed across different centers and scanners. Although stratified analyses were performed, the potential impact of inter-scanner variability and the absence of central image review should be discussed in greater detail.

We acknowledge this limitation: although all PET/CT devices were EARL-certified and analyses were stratified by center to account for inter-scanner heterogeneity (including analog versus digital technology), images were not centrally reviewed, and cross-calibration across the four devices was not feasible within the pragmatic, real-world design of this multicenter study, which may have introduced residual quantitative variability in [18F]FDG uptake measurements not fully captured by center-stratified Z-scores.

Minor Comments

Several typographical and grammatical errors should be corrected throughout the manuscript.

We thank the Reviewer #1 for highlighting the need for improved clarity and correction of typographical errors. Upon careful re-reading, we identified and corrected the following in the final version: (1) "vriemia" / "viremia" (Patients and Methods); (2) "rejction" / "rejection" (Introduction, ref. 12-15); (3) inconsistent spacing in "postKTx" / "post KTx" throughout the manuscript; (4) unclosed brackets in several 95%CI values (e.g., "OR=1.542 [1.021–2.328, p=0.04)" missing closing bracket, occurring at multiple instances); (5) unclosed parentheses in Table 1 ("9 (100.0" for MMF and DSA "None" rows); (6) extraneous space in "OR= 1.051"; (7) inconsistent naming: "Universitair Ziekenhuis Antwerpen" (main text) versus "Universiteit Ziekenhuis Antwerpen" (Supplementary Figure 1 legend); (8) inconsistent terminology, "biopsy-proven AR" (Statistics section) versus "SCR" used elsewhere; (9) minor rounding discrepancy, p=0.07 (Abstract) versus p=0.066 (Results), for the same mSUVR OR; and (10) inconsistent spacing, "CHULiège" versus "CHU Liège" (Supplementary Table 1 legend). All these points have been corrected in the revised manuscript to ensure internal consistency and compliance with PLOS ONE editorial standards.

The rationale for combining borderline lesions and rejection lesions in some analyses should be explained more clearly.

The biopsy-proven SCR group was small (n=9, including 6 TCMR and 3 MVI), despite our cohort of 185 patients exceeding the pre-specified sample size (n=156) calculated to detect a 7% SCR incidence with 80% power. This limited event count restricts the precision of estimates specific to the SCR subgroup, with wide confidence intervals around mSUVR values and diagnostic performance metrics. To partly address this, we combined SCR with borderline cases (n=27, i.e. 14.6%) for the primary multivariate analysis of "non-normal histology," which improved statistical power and allowed significant associations to emerge (e.g., Z-score of mSUVR, OR=1.542, p=0.04). Still, we have to admit that 5/6 TCMR showed mSUVR below the 2.4 threshold established by our previous study. We now explicitly acknowledge in the Discussion that the low SCR incidence, while consistent with contemporary cohorts, limits definitive conclusions regarding mSUVR's diagnostic yield specifically for SCR, and larger multicenter cohorts are needed for adequately powered SCR-specific analyses.

Figure legends could be expanded to improve clarity and facilitate interpretation without referring extensively to the main text.

We respectfully disagree with Reviewer #1.

The discussion may benefit from a brief comparison with emerging non-invasive biomarkers such as donor-derived cell-free DNA and molecular diagnostics.

The revised version of the Discussion refers to a recent review in the field: Cardoso de Aguiar R, Willicombe M, Roufosse C. Noninvasive Diagnosis of Kidney Allograft Rejection. J Am Soc Nephrol. 2025 Nov;36(11):2269–85.

Reviewer #2: (No Response)

We thank Reviewer #2 Massimo CASTELLANI for confirming that all comments and suggestions have been adequately addressed.

________________________________________

7. PLOS authors have the option to publish the peer review history of their article (what does this mean?). If published, this will include your full peer review and any attached files.

If you choose “no”, your identity will remain anonymous but your review may still be made public.

Do you want your identity to be public for this peer review? For information about this choice, including consent withdrawal, please see our Privacy Policy.

Reviewer #1: No

Reviewer #2: Yes: Massimo CASTELLANI

________________________________________

# Peer review: Decision Letter 2

**DOI:** 10.1371/journal.pone.0345426.r005

**Authors:** Sethu Subha

13 Aug 2026

A multicenter prospective validation cohort does not support the use of kidney/psoas [18F]FDG uptake in the diagnosis of kidney allograft subclinical rejection

PONE-D-26-10474R2

Dear Dr.

We’re pleased to inform you that your manuscript has been judged scientifically suitable for publication and will be formally accepted for publication once it meets all outstanding technical requirements.

Within one week, you’ll receive an e-mail detailing the required amendments. When these have been addressed, you’ll receive a formal acceptance letter and your manuscript will be scheduled for publication.

An invoice will be generated when your article is formally accepted. Please note, if your institution has a publishing partnership with PLOS and your article meets the relevant criteria, all or part of your publication costs will be covered. Please make sure your user information is up-to-date by logging into Editorial Manager at Editorial Manager® and clicking the ‘Update My Information' link at the top of the page. For questions related to billing, please contact billing support.

If your institution or institutions have a press office, please notify them about your upcoming paper to help maximize its impact. If they’ll be preparing press materials, please inform our press team as soon as possible -- no later than 48 hours after receiving the formal acceptance. Your manuscript will remain under strict press embargo until 2 pm Eastern Time on the date of publication. For more information, please contact onepress@plos.org.

Kind regards,

Sethu Thakachy Subha, M.S

Academic Editor

PLOS One

Additional Editor Comments (optional):

Reviewers' comments:

Reviewer's Responses to Questions

**Comments to the Author**

Reviewer #1: All comments have been addressed

**********

2. Is the manuscript technically sound, and do the data support the conclusions?

Reviewer #1: Yes

**********

3. Has the statistical analysis been performed appropriately and rigorously?

Reviewer #1: N/A

**********

4. Have the authors made all data underlying the findings in their manuscript fully available?

The PLOS Data policy

Reviewer #1: Yes

**********

5. Is the manuscript presented in an intelligible fashion and written in standard English?

Reviewer #1: Yes

**********

Reviewer #1: Corrections. Made optimally.

Acceptance of the case with your case update.

Topic can be explored further for high number pts subsequently

**********

what does this mean?). If published, this will include your full peer review and any attached files.

If you choose “no”, your identity will remain anonymous but your review may still be made public.

**Do you want your identity to be public for this peer review?**  For information about this choice, including consent withdrawal, please see our Privacy Policy

Reviewer #1: **Yes:** Prof Dr Sunita Nitin Sonavane

**********

# Editor report: Acceptance letter

**DOI:** 10.1371/journal.pone.0345426.r006

**Authors:** Sethu Subha

PONE-D-26-10474R2

PLOS One

Dear Dr. Jouret,

I'm pleased to inform you that your manuscript has been deemed suitable for publication in PLOS One. Congratulations! Your manuscript is now being handed over to our production team.

At this stage, our production department will prepare your paper for publication. This includes ensuring the following:

* All references, tables, and figures are properly cited

* All relevant supporting information is included in the manuscript submission,

* There are no issues that prevent the paper from being properly typeset

You will receive further instructions from the production team, including instructions on how to review your proof when it is ready. Please keep in mind that we are working through a large volume of accepted articles, so please give us a few days to review your paper and let you know the next and final steps.

Lastly, if your institution or institutions have a press office, please let them know about your upcoming paper now to help maximize its impact. If they'll be preparing press materials, please inform our press team within the next 48 hours. Your manuscript will remain under strict press embargo until 2 pm Eastern Time on the date of publication. For more information, please contact onepress@plos.org.

You will receive an invoice from PLOS for your publication fee after your manuscript has reached the completed accept phase. If you receive an email requesting payment before acceptance or for any other service, this may be a phishing scheme. Learn how to identify phishing emails and protect your accounts at https://explore.plos.org/phishing.

If we can help with anything else, please email us at customercare@plos.org.

Thank you for submitting your work to PLOS One and supporting open access.

Kind regards,

PLOS One Editorial Office Staff

on behalf of

Dr. Sethu Thakachy Subha

Academic Editor

PLOS One
